# RORα–GABP–TFAM axis alleviates myosteatosis with fatty atrophy through reinforcement of mitochondrial capacity

**DOI:** 10.1002/jcsm.13432

**Published:** 2024-01-25

**Authors:** Hyeon‐Ji Kim, Sang‐Heon Lee, Cheolhee Jeong, Yong‐Hyun Han, Mi‐Ock Lee

**Affiliations:** ^1^ College of Pharmacy Seoul National University Seoul South Korea; ^2^ Research Institute of Pharmaceutical Sciences Seoul South Korea; ^3^ College of Pharmacy Kangwon National University Chuncheon South Korea; ^4^ Bio‐MAX Institute Seoul National University Seoul South Korea

**Keywords:** fatty atrophy, mitochondrial biogenesis, myosteatosis, NAFLD, RORα

## Abstract

**Background:**

Fat infiltration in muscle, called ‘myosteatosis’, precedes muscle atrophy, which subsequently results in sarcopenia. Myosteatosis is frequently observed in patients with nonalcoholic fatty liver disease (NAFLD). We have previously reported that retinoic acid receptor‐related orphan receptor‐α (RORα) regulates mitochondrial dynamics and mitophagy in hepatocytes, resulting in an alleviation of NAFLD. In this study, we aimed to investigate the role of RORα in skeletal muscle and to understand molecular mechanisms by which RORα controls mitochondrial capacity, using an NAFLD‐associated myosteatosis mouse model.

**Methods:**

To establish a myosteatosis model, 7‐week‐old C57BL/6N mice were fed with high‐fat diet (HFD). After 15 weeks of diet feeding, an adeno‐associated virus vector encoding RORα (AAV‐RORα) was injected to gastrocnemius (GA) muscles, or after 7 weeks of HFD feeding, JC1‐40, an RORα agonistic ligand, was administered daily at a dose of 5 mg/kg/day by oral gavage for 5 weeks. Histological, biochemical and molecular analyses in various in vivo and in vitro experiments were performed.

**Results:**

First, the number of oxidative MyHC2a fibres with intensive lipid infiltration increased by 3.8‐fold in the red region of the GA of mice with myosteatosis (*P* < 0.001). RORα was expressed around MyHC2a fibres, and its level increased by 2.7‐fold after HFD feeding (*P* < 0.01). Second, treatment of RORα ligands in C2C12 myoblasts, such as cholesterol sulfate and JC1‐40, enhanced the number of oxidative fibres stained for MyHC1 and MyHC2a by two‐fold to four‐fold (*P* < 0.01), while it reduced the lipid levels in MyHC2a fibres by 20–50% (*P* < 0.001) in the presence of palmitic acids. Third, mitochondrial membrane potential (*P* < 0.01) and total area of mitochondria (*P* < 0.01) were enhanced by treatment of these ligands. Chromatin immunoprecipitation analysis showed that RORα bound the promoter of GA‐binding protein α subunit gene that led to activation of mitochondrial transcription factor A (TFAM) in C2C12 myoblasts (*P* < 0.05). Finally, intramuscular transduction of AAV‐RORα alleviated the HFD‐induced myosteatosis with fatty atrophy; lipid contents in MyHC2a fibres decreased by 48% (*P* < 0.001), whereas the number of MyHC2b fibre increased by 22% (*P* < 0.001). Also, administration of JC1‐40 improved the signs of myosteatosis in that it decreased the level of adipose differentiation‐related protein (*P* < 0.01) but increased mitochondrial proteins such as cytochrome *c* oxidase 4 and TFAM in GA muscle (*P* < 0.01).

**Conclusions:**

RORα plays a versatile role in regulating the quantity of mitochondria and the oxidative capacity, ultimately leading to an improvement in myosteatosis symptoms.

## Introduction

Fat infiltration in muscle, called ‘myosteatosis’, precedes muscle atrophy, which subsequently results in sarcopenia. This condition often coexists with chronic metabolic diseases such as nonalcoholic fatty liver disease (NAFLD, also known as metabolic dysfunction‐associated steatotic liver disease), dyslipidaemia, insulin resistance and cardiovascular diseases.^1^ Skeletal muscle plays a key role in regulating metabolism, accounting for 30% to almost 100% of total metabolic activity of the body.^2^ It is a heterogeneous tissue made up of various fibre types, which are broadly classified as type 1 ‘slow‐twitch’ and type 2 ‘fast‐twitch’. Fast‐twitch fibres are further subdivided into 2a, 2x and 2b based on differential myosin heavy chain (MyHC) gene expression. Type 1 and type 2a fibres have a high number of mitochondria and primarily use oxidative metabolism, while type 2x and type 2b fibres have limited numbers of mitochondria and primarily use glycolytic metabolism.^3^ In patients with diabetes or individuals with obesity, the lipid contents in the vastus lateralis were higher by 25–50% than those in lean individuals. Type 1 fibres have the highest concentration of neutral lipids, followed by intermediate levels in type 2a fibres and the lowest levels in type 2b fibres.^4^ In animal models of diet‐induced myosteatosis, intramyocellular lipid (IMCL) build‐up was most prevalent in oxidative fibres undergoing a transition from glycolytic to oxidative fibre type.^5^ NAFLD, most common chronic liver disease, ranges from steatosis to nonalcoholic steatohepatitis (NASH), characterized by increased oxidative stress and lipotoxicity.^6^ Recently, several studies have reported that risk of NASH increased in patients with myosteatosis compared with patients without myosteatosis.^7^, ^8^ In NASH preclinical models, degree of myosteatosis apparently discriminated NASH from benign fatty liver and normal liver.^9^ These observations indicate a close relationship between myosteatosis and NAFLD.

Proper functioning of mitochondria is essential for the oxidative metabolism of lipids in muscle. Any malfunction of these organelles is closely tied to oxidative stress, lipotoxicity and inflammation in muscle cells.^10^ Clinically, mitochondrial enzyme activity and lipid oxidation were reduced in skeletal muscles of patients with obesity relative to those in lean subjects.^4^, ^11^ Biogenesis of mitochondria is regulated by nuclear respiratory factor (NRF) 1 and GA‐binding protein (GABP, also known as nuclear respiratory factor 2), which regulate the expression of a significant number of proteins constituting the respiratory complexes of mitochondria and mitochondrial transcription factor A (TFAM). The functions of NRF1 and GABP are potentiated by interaction with their coactivator, peroxisome proliferator‐activated receptor‐γ coactivator (PGC)‐1α.^12^ In a tenectomy and denervation‐induced myosteatosis model, NRF1 and TFAM were downregulated in the rotator cuff musculature of rat.^13^ The skeletal muscles of patients with obesity and diabetes displayed a decrease in the expression of PGC‐1α and PGC‐1α‐controlled mitochondrial genes. This reduction was accompanied by the accumulation of ectopic triglycerides (TGs).^14^ These observations suggest that the activity of mitochondria in oxidative fibres is critical for reducing intramuscular lipids and improving muscle quality in conditions of myosteatosis.

Retinoic acid receptor‐related orphan receptor‐α (RORα) is a ligand‐dependent transcription factor that belongs to the steroid hormone receptor superfamily.^15^ It has been reported that hepatic RORα has a protective role against the progression of NAFLD by increasing mitochondrial function, maintaining homeostasis of hepatic lipid metabolism and resolving inflammation.^16^, ^17^, ^18^, ^19^ The potential role of RORα in skeletal muscle was first identified following an observation that muscular atrophy was present in the RORα‐deficient staggerer mice (RORα sg/sg), which expressed a C‐terminal deletion form of RORα.^20^ RORα promoted muscle differentiation through direct interaction with transcriptional coactivators such as p300 and myoD in differentiating C2C12 cells.^21^ Furthermore, ectopic expression of a dominant negative RORα in skeletal muscle cells attenuated expression of many genes involved in lipid homeostasis, such as muscle‐type carnitine palmitoyltransferase‐1 and caveolin‐3.^22^ The administration of nobiletin, an RORα activator, restored RORα expression and increased mitochondrial respiratory chain complex activity in skeletal muscles.^23^ A natural compound, baicalein, upregulated the expression and secretion of fibroblast‐growth factor 21 via an RORα‐dependent manner in C2C12 myotubes.^24^ These observations suggest that muscle quality can be improved by activating muscular RORα, which leads to alleviation of symptoms of metabolic diseases including NAFLD. Therefore, we aimed to investigate the role of RORα in skeletal muscle, especially that associated with NAFLD, and to understand molecular mechanisms by which RORα controls mitochondrial capacity, using a mouse model of NAFLD‐associated myosteatosis.

## Methods

### Animal experiments

To establish a diet‐induced myosteatosis model, 7‐week‐old C57BL/6N male mice were fed with a low‐fat diet (LFD; D12450J; Research Diets, New Brunswick, NJ, USA) or a high‐fat diet (HFD; D12492; Research Diets), which contains 60 kcal% fat in the form of lard and soybean oil for 20 weeks. The LFD was made isocaloric to the HFD by adding an ingredient in the form of Lodex 10. After 15 weeks of diet feeding, an adeno‐associated virus (AAV)‐empty vector (EV) or AAV‐RORα (2 × 10^11^ genome copies/20 μL) was injected to right gastrocnemius (GA) muscles, or after 7 weeks of LFD or HFD feeding, JC1‐40, an RORα agonistic ligand, was suspended in 0.5% carboxymethyl cellulose and administered daily at a dose of 5 mg/kg/day by oral gavage for 5 weeks. JC1‐40 was synthesized as described.^25^ GA tissues were dissected from the hind limbs of euthanized mice and immediately placed in 30% sucrose (S7903, Sigma‐Aldrich, St. Louis, MO, USA) solution after dissection. After 20 h, GA tissues were embedded in a formulation of glycols and resins (4583, Sakura Finetek, Torrance, CA, USA) and frozen with liquid nitrogen‐cooled isopentane (M32631, Sigma‐Aldrich).

### Cell lines and cell culture

Mouse myoblast C2C12 cells (CRL‐1772, ATCC, Manassas, VA, USA) were grown in Dulbecco's modified Eagle's medium (DMEM) (Hyclone, Logan, UT, USA) containing 10% foetal bovine serum (FBS). To differentiate confluent C2C12 cells to myotubes, cells were cultured in DMEM containing 2% horse serum (Gibco™ 16050‐130, Thermo Fisher Scientific, Waltham, MA, USA). To prepare the bovine serum albumin (BSA) (A8806, Sigma‐Aldrich)‐conjugated fatty acids, the media containing 0.5% BSA and 0.1‐mM palmitic acid (PA) were incubated at 37°C for 1 h. Cholesterol sulfate (C9523, Sigma‐Aldrich), JC1‐40 or PA (P5585, Sigma‐Aldrich) conjugated with BSA was added to the differentiation medium at Day 2 after cell seeding. Synthesis and preparation of JC1‐40 have been described.^25^

### Immunofluorescence and immunohistochemistry

Frozen sections (10 μm) of muscle tissue were prepared from the mid‐belly region of the snap‐frozen GA muscles using a cryostat microtome (CM3050 S, Leica, Wetzlar, Germany), or GA muscle tissues were fixed in 10% neutral buffered formalin (HT501128, Sigma‐Aldrich), embedded in paraffin and cut into 10‐μm sections. C2C12 cells were fixed with ice‐cold methanol for 15 min or 4% formaldehyde at room temperature for 15 min. For immunohistochemistry, sections of snap‐frozen or paraffin‐embedded GA were stained with specific antibodies (*Table* *S1*). For intramuscular lipid staining, sections of GA were stained with 5‐μM BODIPY (D3922, Thermo Fisher Scientific). Stained tissues were examined using an automated multimodal tissue analysis system (Vectra 3, PerkinElmer, Waltham, MA, USA). Immunofluorescence was performed on tissue sections or C2C12 cells using specific antibodies as described (*Table* *S1*).^17^ The stained specimens were then examined using a confocal microscope (TCS SP8FSU, Leica, Wetzlar, Germany).

### Viruses, plasmids and si‐RNAs

Lentiviral vectors encoding sh‐green fluorescent protein (GFP) and sh‐RORα were constructed using pLKO‐TRC (Addgene, Watertown, MA, USA). The lentiviruses were obtained by transient transfection of the lentiviral vectors with psPAX2 packaging plasmids and pMD2.G envelope plasmids into 293FT cells. After 48 h, viral supernatants were concentrated using Lenti‐Con reagent (LGV‐1021, Lugen SCI Co. Ltd, Bucheon, South Korea), and lentiviruses were stored in HIV‐Safe manager solution (LGV‐1022, Lugen SCI Co. Ltd). Titre of the lentiviruses was determined with the Lenti‐X qRT‐PCR kit (631235, Takara, Tokyo, Japan). The production of Ad‐GFP and Ad‐GFP‐RORα was as described.^25^ For transduction of adenovirus, C2C12 cells were seeded in 12‐well plate. After 24 h, purified adenovirus particles of 100 multiplicity of infection (MOI) were added to the culture media and cells were further cultured until harvested. The transduction efficiency is confirmed by GFP fluorescence. The production and transduction of AAV‐EV and AAV‐RORα were as follows: AAV‐RORα encoding RORα was constructed using pAAV‐MCS (VPK‐410, Cell Biolabs, San Diego, CA, USA). HEK293T cells were used for production of AAVs encoding RORα and AAV‐EV. AAVs were purified using iodixanol gradient ultracentrifugation. The production and purification of AAV were performed in the Virus Facility of the Korea Institute of Science and Technology (Seoul, South Korea). AAV‐EV or AAV‐RORα (2 × 10^11^ genome copies/20 μL) was injected to the right GA muscles. The eukaryotic expression vector encoding FLAG‐tagged full length RORα has been described.^25^ The si‐RNA duplexes targeting mouse RORα were synthesized by Bioneer Co. (Daejeon, South Korea) (*Table* *S2*). si‐RNAs were transfected using Lipofectamine 2000 reagent (11668027, Invitrogen, Waltham, MA, USA) according to the manufacturer's protocol.

### Assessment of mitochondrial function and electron microscopy

For succinate dehydrogenase (SDH) staining, sections of snap‐frozen GA were incubated in 0.2‐M sodium phosphate‐buffered solution (pH 7.6) containing 50‐mM sodium succinate (S9637, Sigma‐Aldrich) and 0.6‐mM nitrotetrazolium blue chloride (N6876, Sigma‐Aldrich) at 37°C. After 30 min, slides were washed with distilled water. Tissues were then examined using an automated multimodal tissue analysis system (Vectra 3, PerkinElmer).

For electron microscopy, whole GA or plantaris muscle tissues, or C2C12 cells were fixed in 2.5% glutaraldehyde in 0.1‐M phosphate buffer (pH 7.0). Samples were post‐fixed with osmium tetroxide, followed by en bloc staining with 0.5% uranyl acetate. The samples were then dehydrated using 30%, 50%, 70%, 80%, 90% and 100% ethanol, embedded in Spurr's resin and incubated at 70°C for polymerization of the resin. Ultrathin sections were cut using an ultramicrotome (EM UC7, Leica) and examined using a transmission electron microscope (120 kV; Talos L120C, FEI, Czech Republic).

### Western blotting and quantitative real‐time PCR

Western blotting was performed as described, using specific antibodies^25^ (*Table* *S1*). Proteins were extracted from C2C12 cells and quantified by bicinchoninic acid assay (23225, Thermo Fisher Scientific). After quantification, sample buffer was added and the samples were left to stand for 30 min at 37°C, for the detection of mitochondrially encoded cytochrome *c* oxidase I (MTCO1) proteins. The intensity of western blots was quantified by using ImageJ software. The mRNA expression of genes was determined by real‐time PCR (qPCR) using an ABI StepOnePlus™ Real‐Time PCR System (Applied Biosystems, Foster City, CA, USA) with specific primers (*Table* *S2*). Relative mRNA expression was calculated relative to controls using the 2^−ΔΔCT^ method.^26^

### Study approval

All experiments were performed in a blinded and randomized fashion. The experimental protocols were approved by the Seoul National University Institutional Animal Care and Use Committee (Permission Number SNU‐220211‐1), and all experiments were conducted according to the committee's guidelines.

### Statistical analysis

All analyses were performed using GraphPad Prism software (GraphPad Software, San Diego, CA, USA). Statistical analyses between two groups were conducted using the nonparametric Mann–Whitney *U* test (two‐tailed). When the experiments contain more than two groups, differences between groups were analysed using one‐way analysis of variance (ANOVA). Two‐way ANOVA was used to compare the means of two independent variables or factors from two or more populations. Data are presented as the mean ± SD. Statistical significance was set at *P* < 0.05.

## Results

### RORα expression is enhanced in oxidative‐type muscle fibres in an HFD‐induced myosteatosis mouse model

**Figure 1:**
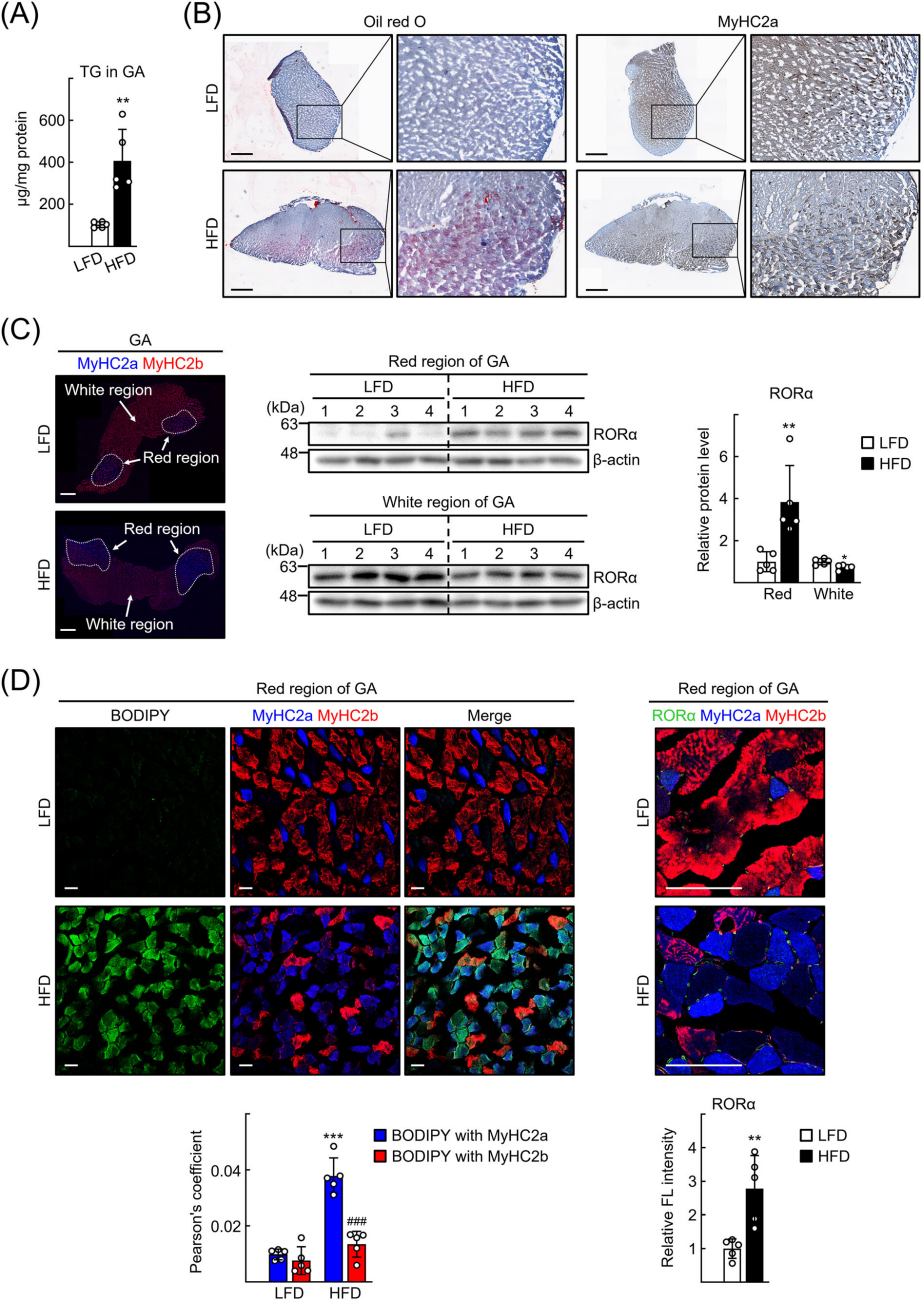
Retinoic acid receptor‐related orphan receptor‐α (RORα) expression level is enhanced in oxidative‐type muscle fibres in a high‐fat diet (HFD)‐induced myosteatosis mouse model. (A) Seven‐week‐old mice were fed with either low‐fat diet (LFD) or HFD for 20 weeks. Triglyceride (TG) contents in gastrocnemius (GA) muscles. TG content was quantified in GA tissues. ^**^ *P* < 0.01 versus LFD (*n* = 5). (B) Oil Red O staining and immunohistochemical staining for MyHC2a of GA tissue sections. Representative images are shown. Scale bar: 1 mm. (C) Mouse GA tissues were subjected to immunostaining for MyHC2a (blue) and MyHC2b (red). Protein lysates of red region and white region of GA tissues were prepared from LFD‐ or HFD‐fed mice. Expression level of RORα was analysed by western blotting. The number represents the relative protein level of RORα when the level of LFD‐fed mouse was considered as 1. Scale bar: 1 mm. *P < 0.05 and ^**^ *P* < 0.01 versus LFD (*n* = 5). (D) The red region of GA tissue sections of the LFD‐ or HFD‐fed mice was stained using BODIPY for visualization of lipid droplets and was subjected to immunostaining for MyHC2a (blue) and MyHC2b (red). Representative images examined by confocal microscopy are shown (left). Expression of MyHC2a (blue), MyHC2b (red) and RORα (green) was visualized by using immunofluorescence. Representative images are presented (right). For quantification of colocalization, Pearson's correlation coefficient was calculated with the JACoP plugin within ImageJ software from the GA sections of each mouse (*n* = 5). Fluorescence intensity of RORα (green) was quantified in one section from five mice by using ImageJ. Scale bar: 25 μm. ^***^ *P* < 0.001 versus BODIPY with MyHC2a in LFD‐fed mice and ^###^ *P* < 0.001 versus BODIPY with MyHC2a in HFD‐fed mice. ^**^ *P* < 0.01 versus LFD (RORα).

To investigate the role of RORα in the development of myosteatosis, we established an NAFLD‐associated myosteatosis mouse model by feeding mice an HFD for 20 weeks (*Figure* *S1* *A*,*B*). Because the TG content was highest in the lower leg muscles in humans with obesity, and the medial head of the human GA displayed locally intensive fatty infiltration,^27^, ^28^ we focused on GA muscles in this study. Similar to the human cases, the TG content was markedly increased in the mouse GA muscle tissues after HFD feeding (*Figure* *1* *A*). The GA muscle can be divided into red and white regions, which consist mainly of oxidative MyHC2a fibres and glycolytic MyHC2b fibres, respectively.^29^ Oil Red O staining was clearly associated with red region MyHC2a fibres, indicating that lipids accumulated in this region of GA muscle (*Figure* *1* *B*). An immunofluorescence study using antibodies against MyHC2a and MyHC2b clearly showed the red and white regions of GA tissues (*Figure* *1* *C*). The level of RORα protein increased markedly in the red region of GA after HFD feeding, whereas it decreased slightly in the white region (*Figure* *1* *C*). Staining intensities of BODIPY and for MyHC2a increased in the red region of GA muscles after HFD feeding. In particular, the muscle fibres stained with BODIPY and MyHC2a increased dramatically in the red region after HFD feeding, indicating that the number of oxidative muscle fibres containing lipids increased in this NAFLD‐associated myosteatosis model (*Figure* *1* *D*). In the soleus, muscle in the lower legs—which is mainly composed of the oxidative fibres, MyHC1 and MyHC2a—lipids were also accumulated in the MyHC2a fibres (*Figure* *S2*). RORα was expressed around the MyHC2a fibres, probably in the nuclei. The intensity of RORα staining in the red region increased by ~3‐fold after HFD feeding (*Figure* *1* *D*). These observations suggest a potential association of RORα in oxidative‐type muscle fibres with the pathophysiology of NAFLD‐induced myosteatosis.

### RORα accelerates differentiation of oxidative myotubes

**Figure 2:**
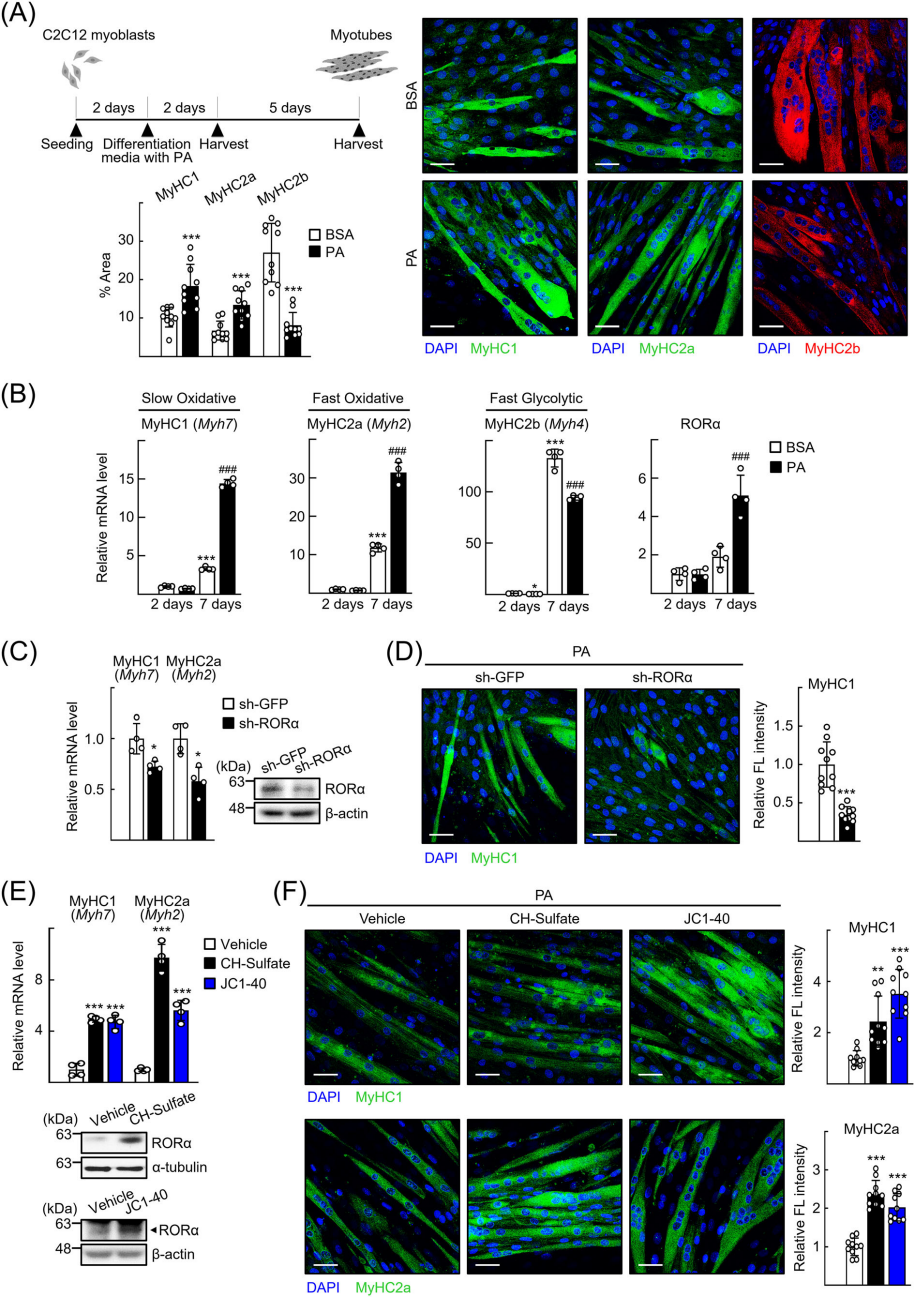
Retinoic acid receptor‐related orphan receptor‐α (RORα) accelerates differentiation of oxidative myotubes in the presence of palmitic acids (PAs). (A) Immunostaining was performed with C2C12 cells in the presence of 0.1‐mM PA conjugated with bovine serum albumin (BSA) at Day 7 of differentiation for staining of MyHC1 (green), MyHC2a (green) or MyHC2b (red), and DAPI (blue). Representative images examined by confocal microscopy are shown. Fluorescent area was quantified in 10 images by using ImageJ software. Scale bar: 25 μm. ^***^ *P* < 0.001 versus BSA. (B) C2C12 cells were incubated in differentiation medium with 0.1‐mM PA conjugated with BSA for 2 or 7 days. Total RNA was isolated, and mRNA levels of the indicated genes were measured by quantitative real‐time PCR (qRT‐PCR). ^***^ *P* < 0.001 versus BSA‐treated C2C12 cells at 2 days and ^###^ *P* < 0.001 versus BSA‐treated C2C12 cells at 7 days (*n* = 4). (C) C2C12 cells were transduced by either lenti‐sh‐GFP or lenti‐sh‐RORα. After 24 h, C2C12 cells were exposed to differentiation medium containing 0.1‐mM PA conjugated with BSA. After 7 days, total RNA was isolated and mRNA levels of the indicated genes were measured by qRT‐PCR. Expression levels of RORα in lentivirus‐transduced C2C12 cells were analysed by western blotting. *P < 0.05 versus lenti‐sh‐GFP‐transduced C2C12 cells (*n* = 4). (D) Immunostaining was performed with either lenti‐sh‐GFP‐ or lenti‐sh‐RORα‐transduced C2C12 myotubes in the presence of 0.1‐mM PA conjugated with BSA at Day 7 of differentiation for staining of MyHC1 (green). C2C12 myotubes stained for MyHC1 were examined by confocal microscopy. Representative images are shown. Data were obtained from three independent experiments, and fluorescence intensity was quantified in 10 images of each group by using ImageJ. Scale bar: 25 μm. ^***^ *P* < 0.001 versus lenti‐sh‐GFP‐transduced C2C12 cells. (E) C2C12 cells were exposed to differentiation medium containing 0.1‐mM PA conjugated with BSA and treated with cholesterol sulfate (CH‐sulfate) or JC1‐40. After 7 days, total RNA was isolated and mRNA levels of the indicated genes were measured by qRT‐PCR. Expression levels of RORα in C2C12 myotubes treated with CH‐sulfate or JC1‐40 were analysed by western blotting. ^***^ *P* < 0.001 versus vehicle (*n* = 4). (F) Immunostaining was performed with CH‐sulfate or JC1‐40‐treated C2C12 myotubes for staining of MyHC1 (green) or MyHC2a (green) and DAPI (blue). Representative images examined by confocal microscopy are shown. Data were obtained from three independent experiments, and fluorescence intensity was quantified in 10 images of each group by using ImageJ. Scale bar: 25 μm. ^**^ *P* < 0.01 and ^***^ *P* < 0.001 versus vehicle.

Therefore, we examined whether RORα has a role in the differentiation of oxidative muscle fibres, particularly in a lipid‐rich environment using C2C12 myoblasts. When C2C12 myoblasts were treated with media inducing differentiation, differentiated myotubes stained for MyHC1 (encoded by the Myh7 gene), MyHC2a (encoded by the Myh2 gene) and MyHC2b (encoded by the Myh4 gene), markers for slow oxidative, fast oxidative and fast glycolytic fibres, respectively, were observed at Day 7 of differentiation. Treatment with PAs further increased the number of fibres stained for MyHC1 and MyHC2a but decreased staining for MyHC2b (*Figure* *2* *A*). Patterns of Myh7, Myh2 and Myh4 mRNA expression displayed a similar profile. Interestingly, RORα mRNA levels increased in a pattern similar to that of oxidative muscle fibres (*Figure* *2* *B*). Knockdown of RORα decreased the level of Myh7 and Myh2 mRNA expression and the level of MyHC1 protein in the PA‐treated C2C12 myotubes (*Figure* *2* *C*,*D*). In contrast, transduction of an adenovirus encoding RORα (Ad‐RORα) enhanced the levels of Myh7 and Myh2 expression (*Figure* *S3*). Treatment with activating ligands of RORα, such as cholesterol sulfate (CH‐sulfate) and JC1‐40, further enhanced the expression of Myh7 and Myh2 mRNA and the number of fibres stained for MyHC1 and MyHC2a (*Figure* *2* *E*,*F*). After PA treatment, accumulation of lipids was observed in the myotubes stained for MyHC2a, as observed in the red region of GA of mice with myosteatosis, and the lipid levels were reduced by treatment with the RORα‐activating ligands (*Figure* *S4A*). Overexpression of RORα also blocked lipid accumulation in myotubes (*Figure* *S4B*). Together, these results indicate that RORα has a role in inducing differentiation of oxidative myotubes in lipid‐rich conditions and that it blocks further accumulation of lipids in oxidative myotubes.

### RORα enhances mitochondrial mass and function in PA‐treated C2C12 cells

**Figure 3:**
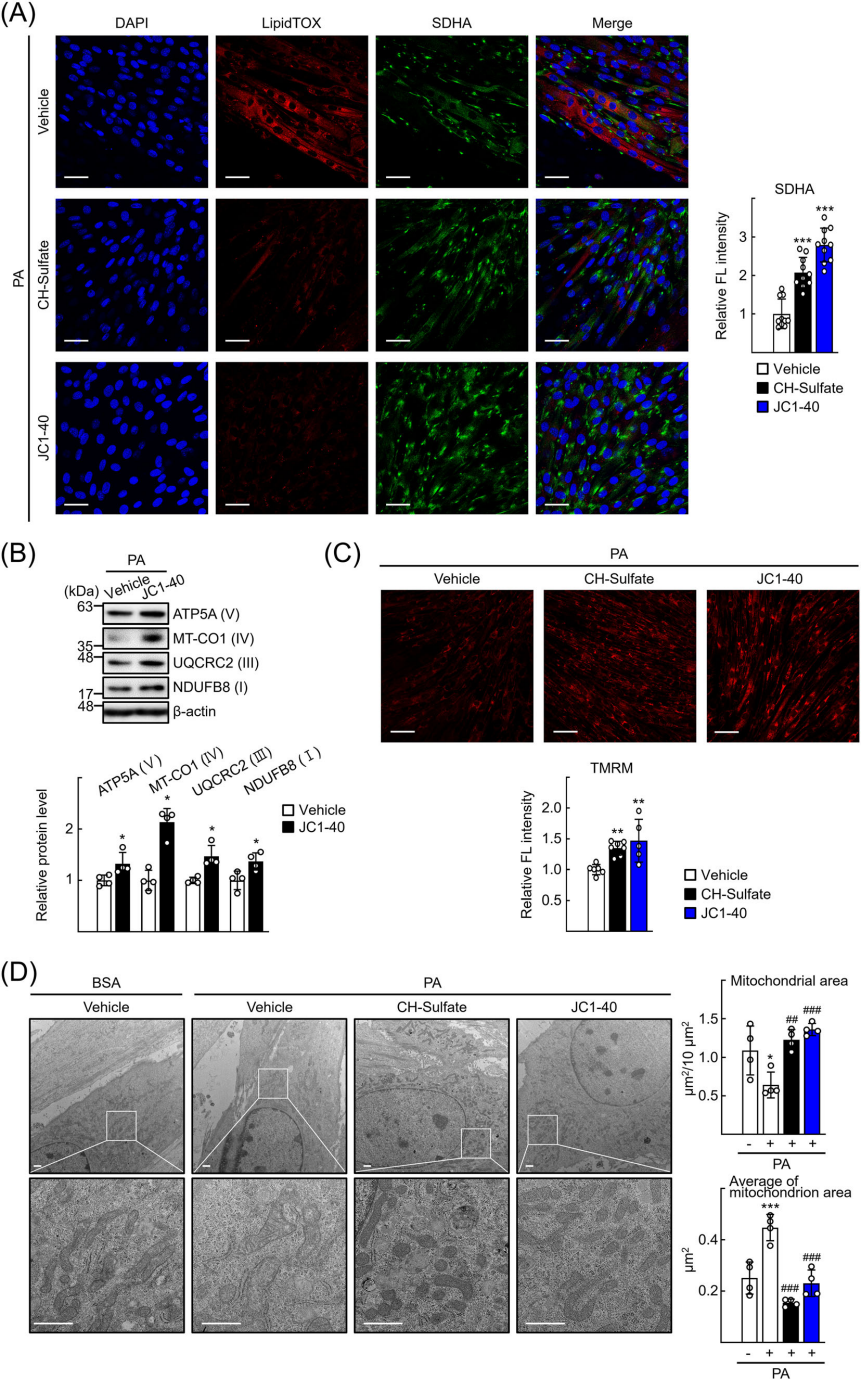
Retinoic acid receptor‐related orphan receptor‐α (RORα) enhances mitochondrial function and rescues mitochondria mass in palmitic acid (PA)‐challenged C2C12 cells. (A) C2C12 cells were exposed to differentiation medium containing 0.1‐mM PA conjugated with bovine serum albumin (BSA) and treated with cholesterol sulfate or JC1‐40. After 7 days, immunostaining was performed for staining of succinate dehydrogenase A subunit (SDHA) (green). C2C12 myotubes were stained with LipidTOX Red for staining of neural lipids and examined by confocal microscopy. Representative images are shown. Data were obtained from three independent experiments, and fluorescence intensity was quantified in 10 images of each group by using ImageJ. Scale bar: 25 μm. ^***^ *P* < 0.001 versus vehicle. (B) C2C12 cells were incubated in differentiation medium with 0.1‐mM PA conjugated with BSA and treated with JC1‐40 20 μM for 2 days. Expression levels of OXPHOS proteins in electron transport chain (ETC) complexes were analysed by western blotting using a commercially available anti‐total OXPHOS primary antibody cocktail. ATP5A, ATP synthase, H^+^ transporting, mitochondrial F1 complex, alpha 1; MTCO1, mitochondrially encoded cytochrome *c* oxidase I; NDUFB8, NADH dehydrogenase (ubiquinone) 1 beta subcomplex subunit 8; UQCRC2, cytochrome *b*–*c*1 complex subunit 2. Roman numbers represent the corresponding ETC complex. Band intensities of each protein were quantified using ImageJ and normalized to that of β‐actin band. *P < 0.05 versus vehicle (*n* = 4). (C) C2C12 cells were grown in differentiation medium with 0.1‐mM PA conjugated with BSA and treated with cholesterol sulfate (CH‐sulfate) or JC1‐40. After 48 h, cells were stained with tetramethylrhodamine, methyl ester (TMRM) and subjected to confocal microscopy. Data were obtained from three independent experiments, and fluorescence intensity was quantified at least five images of each group using ImageJ. Scale bar: 50 μm ^**^ *P* < 0.01 versus vehicle. (D) C2C12 cells were grown in differentiation medium with 0.1‐mM PA conjugated with BSA and treated with cholesterol sulfate (CH‐sulfate) or JC1‐40 for 2 days. At the end of experiments, cells were fixed in 2.5% glutaraldehyde. Representative electron microscopy (EM) images are shown. Quantification of total mitochondrial area and average of mitochondrion area was performed by using ImageJ. Scale bar: 1 μm. *P < 0.05 and ^***^ *P* < 0.001 versus vehicle with BSA and ^##^ *P* < 0.01 and ^###^ *P* < 0.001 versus vehicle with PA.

We previously demonstrated that RORα plays a crucial role in mitochondria dynamics and fatty acid oxidation, especially in hepatocytes.^16^, ^25^ Thus, we hypothesized that RORα‐mediated mitochondria function may reduce lipid accumulation in skeletal muscle in NAFLD‐associated myosteatosis. To explore this hypothesis, we first evaluated the volume of mitochondria by staining of SDH A subunit (SDHA) after treatment with CH‐sulfate or JC1‐40. These ligands reduced the amount of lipids and increased SDHA expression in PA‐treated C2C12 myotubes (*Figure* *3* *A*). Expression levels of mitochondrial OXPHOS proteins, such as ATP synthase, H^+^ transporting, mitochondrial F1 complex, alpha 1, mitochondrially encoded cytochrome c oxidase I, cytochrome *b*–*c*1 complex subunit 2, and NADH dehydrogenase (ubiquinone) 1 beta subcomplex subunit 8, were increased in JC1‐40‐treated C2C12 cells (*Figure* *3* *B*). Treatment with CH‐sulfate or JC1‐40 increased not only the volume of mitochondria stained by MitoTracker but also mitochondrial membrane potential as indicated by tetramethylrhodamine, methyl ester (TMRM) dye staining (*Figures* *3* *C* and *S5A*). Electron microscopy revealed that most of the mitochondria in the C2C12 cells were deformed and swollen and the total area of mitochondria was reduced after PA treatment. Treatment with CH‐sulfate or JC1‐40 increased the total area of mitochondria and reduced the individual mitochondrion area in PA‐treated C2C12 cells. The rounded mitochondria morphology may suggest that the RORα‐activating ligands induced mitochondrial fission in the C2C12 cells (*Figure* *3* *D*). Transduction of Ad‐RORα significantly increased the maximal oxygen consumption rate (OCR) in C2C12 cells (*Figure* *S5B*). Consistently, in the HFD‐fed mice, expression of cytochrome *c* oxidase 4 (COX4) and SDH activity were markedly reduced in the red region of GA tissues. RORα expression was also enhanced, which may be due to a compensatory mechanism to aim restoration of mitochondrial function (*Figure* *S5C*). Together, these data indicate that muscular RORα enhances the oxidative function of mitochondria and thereby reduces the lipid content of oxidative muscle, especially when lipids are in oversupply.

**Figure 4:**
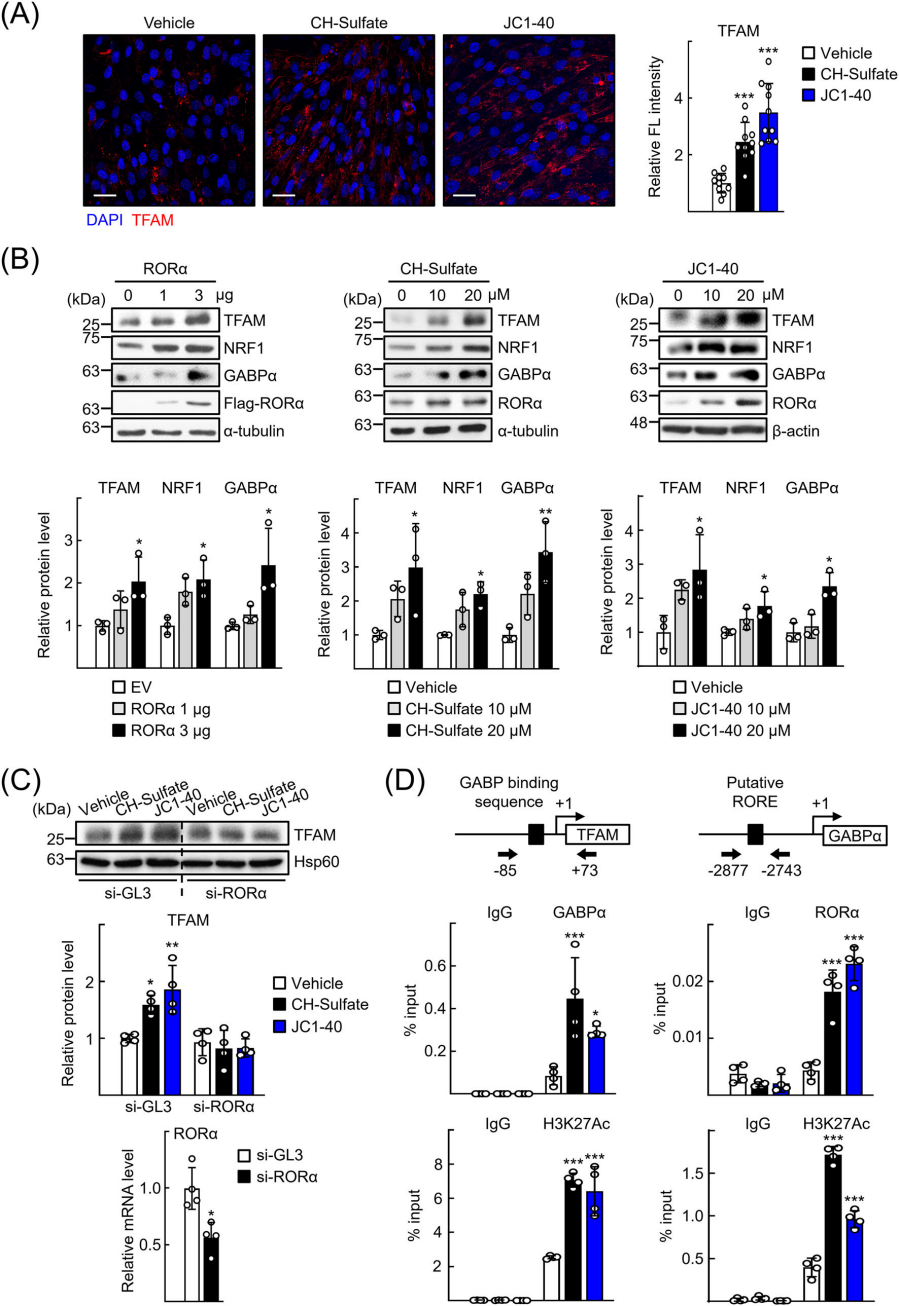
Retinoic acid receptor‐related orphan receptor‐α (RORα) induces the transcription of mitochondrial genes including GA‐binding protein α subunit (GABPα) and mitochondrial transcription factor A (TFAM). (A) Expression of TFAM in the differentiating C2C12 cells treated with cholesterol sulfate (CH‐sulfate) or JC1‐40 was visualized by immunofluorescence. Representative images are presented. Data were obtained from three independent experiments, and fluorescence intensity was quantified in 10 images of each group by using ImageJ. Scale bar: 25 μm. ^***^ *P* < 0.001 versus vehicle. (B) C2C12 cells were transfected with empty vector (EV) or the expression vector encoding Flag‐RORα (RORα) or treated with CH‐sulfate or JC1‐40 in a dose‐dependent manner. Protein levels of Flag‐RORα were analysed by western blotting using anti‐Flag antibody as control. Densitometry was performed using ImageJ software, and protein levels of TFAM, NRF1 and GABPα were normalized to that of α‐tubulin or β‐actin. *P < 0.05 and ^**^ *P* < 0.01 versus EV or vehicle (*n* = 3). (C) C2C12 cells were transfected by si‐GL3 or si‐RORα and then treated with CH‐sulfate or JC1‐40 in differentiation medium for 48 h. mRNA level of RORα was measured by quantitative real‐time PCR (qRT‐PCR) (*n* = 4), and the protein level of TFAM was analysed by western blotting. *P < 0.05 and ^**^ *P* < 0.01 versus si‐GL3 with vehicle (*n* = 4). (D) C2C12 cells were treated with CH‐sulfate or JC1‐40 20 μM in differentiation medium for 48 h. DNA fragments encoding promoter of TFAM gene (GA‐binding protein [GABP] binding sequence: GACCGGAAGTCC) or the regulatory region of GABPα gene (putative RORE: AGGATCTAGGTCAA) were immunoprecipitated with the anti‐GABPα, anti‐H3K27Ac or anti‐RORα antibodies and then amplified by qPCR with specific primers. *P < 0.05 and ^***^ *P* < 0.001 versus vehicle (*n* = 4).

Because TFAM and NRF1/2 are the major transcription regulators of mitochondrial biogenesis, we examined whether these factors were involved in the RORα‐mediated induction of mitochondrial mass.^12^ Treatment with CH‐sulfate or JC1‐40 increased the level of TFAM expression at Day 2 of the early differentiation period (*Figure* *4* *A*). Activation of RORα by either transient overexpression or treatment with CH‐sulfate or JC1‐40 increased the level of TFAM, NRF1 and GABP α subunit (GABPα) expression (*Figure* *4* *B*). The mRNA level of PGC‐1α was also increased after RORα overexpression or JC1‐40 treatment (*Figure* *S6*). Knockdown of RORα by si‐RNA abolished the increase of TFAM, indicating that the effects of CH‐sulfate or JC1‐40 were RORα‐dependent (*Figure* *4* *C*). A chromatin immunoprecipitation (ChIP) assay demonstrated that DNA binding of GABP as well as that of H3K27Ac, an activation marker, to the known regulatory region of the TFAM promoter was enhanced in the presence of CH‐sulfate or JC1‐40.^30^ Using an in silico analysis, we identified a putative RORα response element in the 5′‐upstream region of the GABPα gene. ChIP analysis revealed that RORα bound to the ROR response element in the presence of ligands, suggesting that RORα induces the transcriptional induction of GABPα genes that leads to activation of TFAM and subsequent mitochondrial biogenesis (*Figure* *4* *D*).

### Activation of RORα alleviates myosteatosis with fatty atrophy in HFD‐fed mice

**Figure 5:**
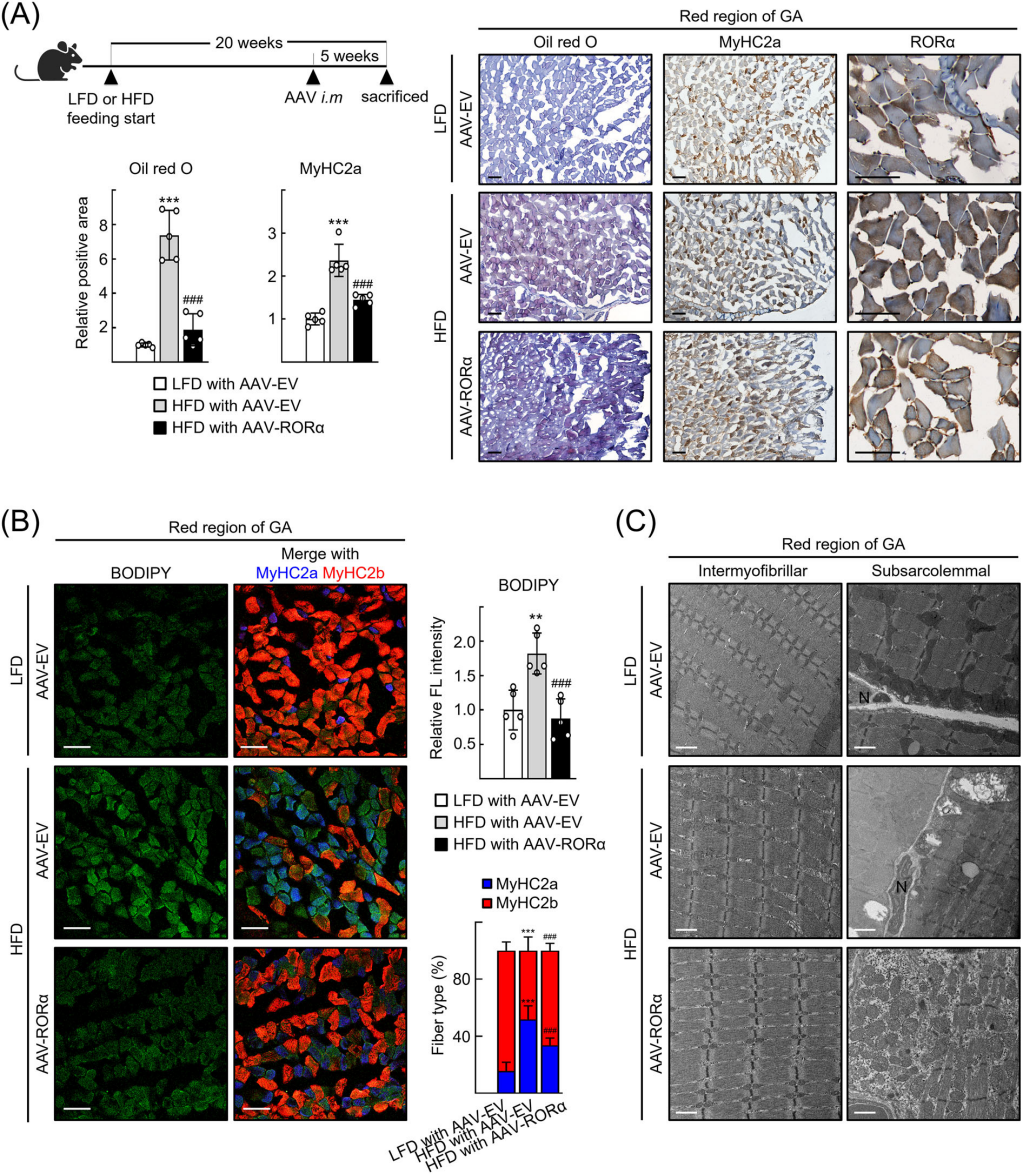
Retinoic acid receptor‐related orphan receptor‐α (RORα) overexpression alleviates myosteatosis in the high‐fat diet (HFD)‐fed mice by reducing lipid accumulation and enhancing mitochondrial biogenesis in oxidative muscle cells. (A) Seven‐week‐old wild‐type mice were fed with low‐fat diet (LFD) or HFD for 20 weeks. At 15 weeks of diet feeding, AAV9‐empty vector (EV) or AAV9‐RORα (2 × 10^11^ genome copies/20 μL) was injected to right gastrocnemius (GA) muscles. Lipid accumulation in red region of GA tissues was assessed by Oil Red O staining. Expression of MyHC2a and RORα in the red region of GA sections of the HFD‐fed and AAV‐RORα‐treated mouse model was visualized by immunohistochemistry. Representative images examined by confocal microscopy are shown. Quantification of Oil Red O staining and MyHC2a‐positive fibres was performed by using ImageJ. Scale bar: 100 μm. ^***^ *P* < 0.001 versus LFD‐fed and AAV‐EV‐treated mouse. ^###^ *P* < 0.001 versus HFD‐fed and AAV‐EV‐treated mouse (*n* = 5). (B) Mouse GA tissues were stained using BODIPY for visualization of lipid droplets and were subjected to immunostaining for MyHC2a (blue) and MyHC2b (red). Representative images examined by confocal microscopy are shown. Green fluorescence intensity and the percentage of blue and red fluorescent area were quantified in five mice using ImageJ. Scale bar: 50 μm. ^**^ *P* < 0.01 and ^***^ *P* < 0.001 versus LFD‐fed and AAV‐EV‐treated mouse and ^###^ *P* < 0.001 versus HFD‐fed and AAV‐EV‐treated mouse (*n* = 5). (C) Representative electron microscopy (EM) images of intermyofibrillar and subsarcolemmal regions of the red region of GA tissues from the HFD‐fed and AAV‐RORα‐treated mouse. Scale bar: 1 μm.

**Figure 6:**
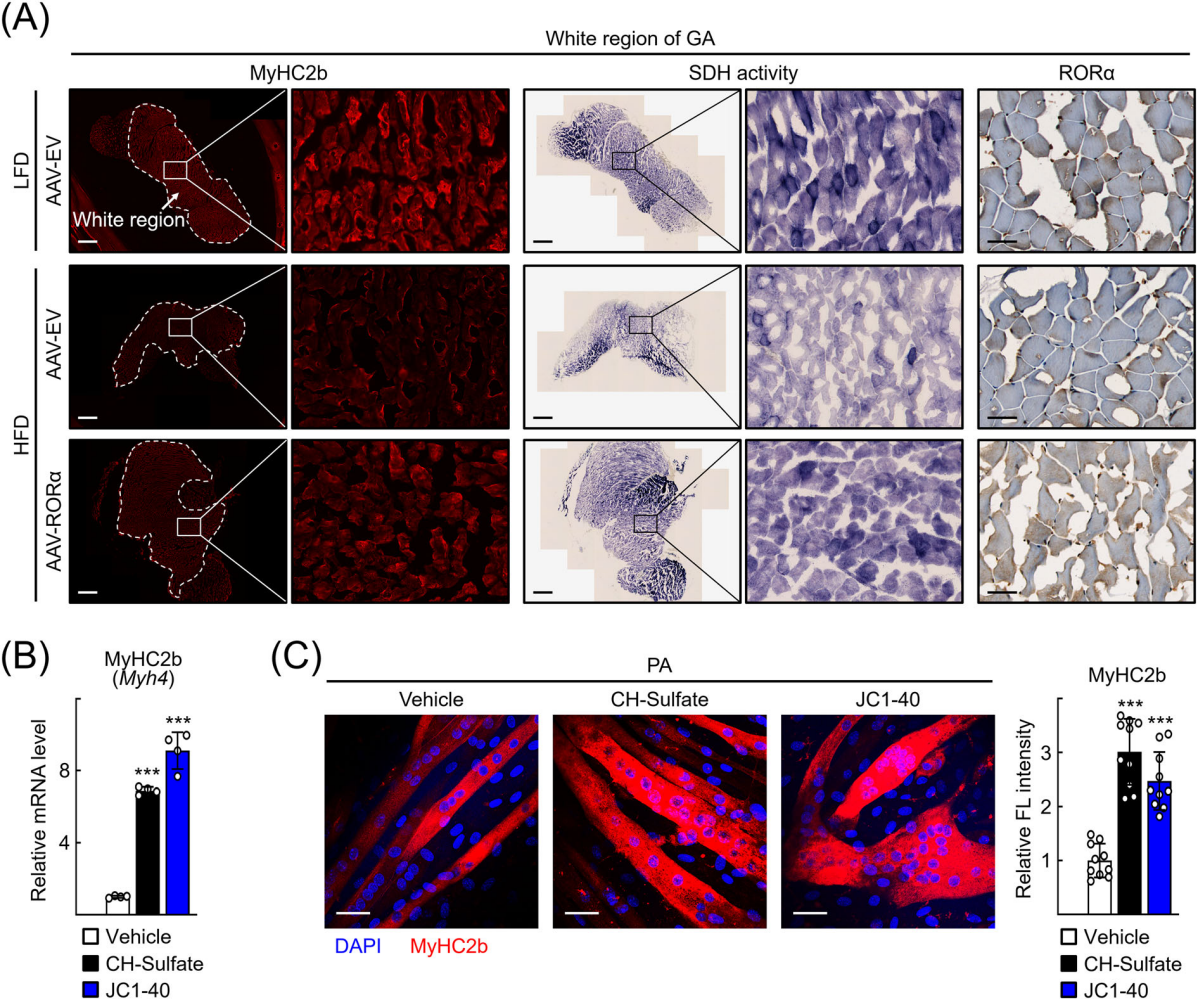
Activation of retinoic acid receptor‐related orphan receptor‐α (RORα) restored the mitochondrial function in glycolytic fibres. (A) Mouse gastrocnemius (GA) tissues were subjected to immunostaining for MyHC2b (red). Succinate dehydrogenase (SDH) staining was performed in the GA sections of the high‐fat diet (HFD)‐fed and AAV‐RORα‐treated mouse model. Tissues were examined by using an automated multimodal tissue analysis system, and representative images are shown. Scale bar: 1 mm (MyHC2b and SDH activity) or 50 μm (RORα). (B) C2C12 cells were exposed to differentiation medium containing 0.1‐mM palmitic acid (PA) conjugated with bovine serum albumin (BSA) and treated with cholesterol sulfate (CH‐sulfate) or JC1‐40. After 7 days, total RNA was isolated and mRNA level of Myh4 was measured by quantitative real‐time PCR (qRT‐PCR). ^***^ *P* < 0.001 versus vehicle (*n* = 4). (C) Immunostaining was performed with C2C12 cells in the presence of 0.1‐mM PA conjugated with BSA at Day 7 of differentiation for staining of MyHC2b (red) and DAPI (blue). Representative images examined by confocal microscopy are shown. Data were obtained from three independent experiments, and fluorescence intensity was quantified in 10 images of each group by using ImageJ. Scale bar: 25 μm. ^***^ *P* < 0.001 versus vehicle.

Finally, we examined the effects of RORα in vivo by intramuscular injection of an AAV encoding RORα (AAV‐RORα) into the GA muscles of the HFD‐fed mice (*Figure* *5* *A*). At 5 weeks after viral transduction, signs of myosteatosis were improved dramatically; lipid content and the number of oxidative type 2a fibres decreased in the red region of GA muscles. At the same time, the number of glycolytic 2b fibres recovered after AAV‐RORα transduction (*Figure* *5* *A*,*B*). An electron microscopy study revealed that mitochondria were enlarged or swollen in the red region of GA muscle tissues of HFD‐fed mice, whereas the size of individual mitochondrion was reduced after transduction of AAV‐RORα in the intermyofibrillar and subsarcolemmal area of GA muscle sections (*Figure* *5* *C*). Consistent with the results from C2C12 cells, TFAM expression was recovered in the red region after transduction of AAV‐RORα, which probably induced mitochondria mass and function, reflected by increased COX4 protein levels and SDH activity in the MyHC2a fibres of red region, respectively (*Figure* *S7*). Interestingly, the increase in mitochondrial function was also observed in the MyHC2b fibres, which may contribute to recovery of the number of glycolytic MyHC2b fibres that largely decreased after HFD feeding (*Figure* *S7*). A significant reduction in the cross‐sectional area of GA was observed after HFD feeding, but it restored following the transduction of AAV‐RORα, indicating that the RORα‐mediated restoration of glycolytic MyHC2b fibres caused alleviation of fatty atrophy (*Figures* *6* *A* and *S8*).

Similarly, SDH activity, which was lowered after HFD feeding, was restored after AAV‐RORα transduction in the white region, indicating that RORα also has a role in mitochondrial biogenesis in this tissue (*Figure* *6* *A*). This idea was supported by observations from in vitro experiments employing C2C12 cells. CH‐sulfate or JC1‐40 treatment restored the level of Myh4 expression and the number of MyHC2b fibres in the PA‐treated C2C12 cells (*Figure* *6* *B*,*C*).

**Figure 7:**
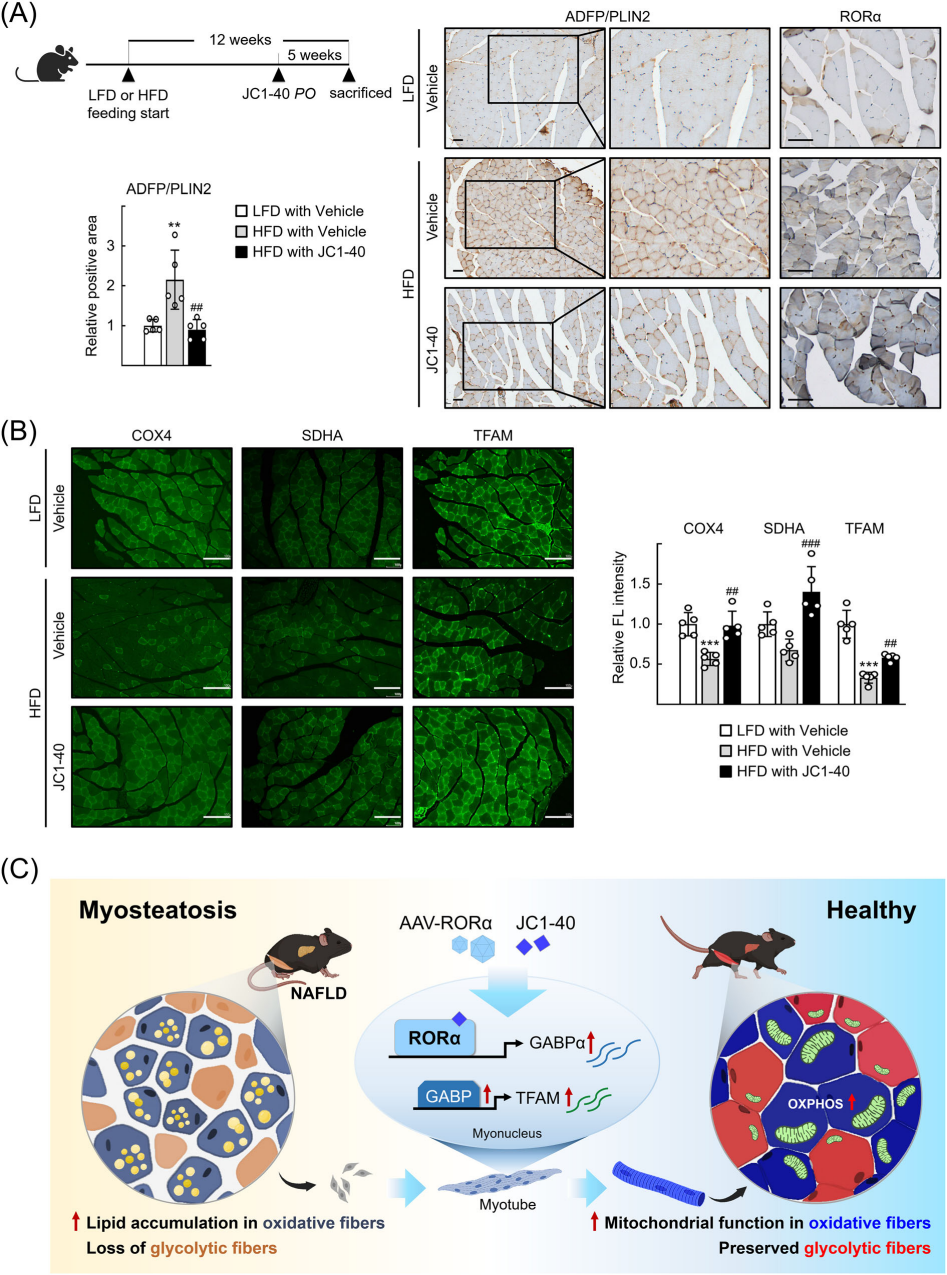
Administration of the retinoic acid receptor‐related orphan receptor‐α (RORα) activator, JC1‐40, attenuates muscular lipid accumulation and rescues mitochondria mass in high‐fat diet (HFD)‐fed mouse model. Wild‐type C57BL/6N mice were fed with either low‐fat diet (LFD) or HFD for 12 weeks. After 7 weeks of diet feeding, JC1‐40 was administered daily at doses of 5 mg/kg/day by oral gavage for 5 weeks. (A) Mouse gastrocnemius (GA) tissues were subjected to immunohistochemistry for staining of adipose differentiation‐related protein, also known as perilipin 2 (ADFP/PLIN2), and RORα. Tissues were examined by using an automated multimodal tissue analysis system, and representative images are presented. Quantification of ADFP/PLIN2‐positive area was performed by using ImageJ. Scale bar: 50 μm. ^**^ *P* < 0.01 versus LFD‐fed and vehicle‐treated mice and ^##^ *P* < 0.01 versus HFD‐fed and vehicle‐treated mice (*n* = 5). (B) Expression of cytochrome *c* oxidase 4 (COX4), succinate dehydrogenase A subunit (SDHA) and mitochondrial transcription factor A (TFAM) in GA sections of the HFD‐fed and JC1‐40‐treated mouse model was visualized by using immunofluorescence. Representative images are presented. Quantification of fluorescence intensity of COX4, succinate dehydrogenase (SDH) and TFAM was performed by using ImageJ. Scale bar: 100 μm. ^***^ *P* < 0.001 versus LFD‐fed and vehicle‐treated mouse (*n* = 5). ^##^ *P* < 0.01 and ^###^ *P* < 0.001 versus HFD‐fed and vehicle‐treated mouse (*n* = 5). (C) Schematic model for the mechanism of RORα‐induced mitochondrial biogenesis in oxidative fibres, leading to attenuation of NAFLD‐associated myosteatosis.

Administration of JC1‐40 also decreased the number of lipid droplets, reflected by a decreased staining intensity of adipose differentiation‐related protein, also known as perilipin 2 (ADFP/PLIN2) proteins. The level of muscular RORα expression, which was increased in the red region of GA during HFD feeding, was further increased by administration of JC1‐40 (*Figure* *7* *A*). The staining intensities of COX4, SDHA and TFAM proteins, which decreased after HFD feeding, were recovered by JC1‐40 administration in the GA muscles (*Figure* *7* *B*). Together, our results indicate that RORα improved the HFD‐induced myosteatosis by enhancing mitochondrial mass and function in GA muscles, which reinforces the differentiation of oxidative muscle cells and oxidative fat catabolism (*Figure* *7* *C*).

## Discussion

Recent studies have revealed that the degree of fat infiltration in skeletal muscle is a reliable indicator of the severity of NAFLD in patients, and myosteatosis is a potential pathophysiological contributor to NASH. In this study, it was discovered that RORα plays a role in enhancing the differentiation and mitochondrial function of oxidative muscle fibres by controlling the expression of TFAM and GABPα. These findings provide insights into a novel regulatory mechanism of RORα in both NAFLD and NAFLD‐associated myosteatosis.

The regulation of mitochondrial dynamics and function in skeletal muscles is achieved through highly precise networks that operate under various energy requirements. Transcriptional factors, such as TFAM, PGC‐1α and p53, control mitochondrial biogenesis by inducing both nuclear‐ and mitochondria‐encoded genes.^31^ TFAM facilitates the transcription of mitochondria‐encoded gene expression and increases copy number and maintains stability of mitochondrial DNA (mtDNA).^32^ TFAM transcription is regulated by the transcriptional coactivator, PGC‐1α, through its interaction with NRFs.^12^ Our study revealed that RORα directly induces transcription of GABPα, which leads to transcriptional induction of TFAM (*Figure* *4* *D*). Furthermore, PGC‐1α was previously demonstrated as a direct downstream target of RORα.^16^ Together, these observations suggest that the RORα–NRF/PGC‐1α–TFAM axis contributes to the restoration of mitochondrial quantity and quality in myosteatotic muscles under NAFLD conditions. p53 also regulates the transcription of TFAM and NRF1 genes, probably via physical interaction between these molecules on the mtDNA.^33^ RORα increased p53 protein level in vascular smooth muscle cells, indicating a potential link between RORα and p53 in the expression of TFAM.^34^ Together, these findings support a pivotal role of RORα in the complex regulatory network of mitochondrial biogenesis and function in muscle.

Skeletal muscle is composed of a distinct combination of oxidative and glycolytic fibres with varying contractile properties and metabolic demands.^3^ A difference in their quantity of mitochondria is an obvious criterion for distinguishing these fibres. Aside from the quantitative difference, each fibre type displays distinct mitochondrial phenotypes characterized by degrees of lipid oxidation capacity, reactive oxygen species‐emitting potential and antioxidant capacity, which are essential to ensure optimal muscle function.^35^ Mitochondrial dynamics, including fission and fusion, is also a distinguishing feature of skeletal muscle fibre types.^36^ Nevertheless, the molecular mechanisms that establish and maintain the different mitochondrial phenotypes in different fibre types remain unclear. In the present study, we discovered that RORα increases the quantity of mitochondria and the lipid oxidation capacity of oxidative MyHC2a fibres, resulting in alleviation of myosteatosis (*Figure* *5* and *S7*). In addition, RORα restored the number of glycolytic MyHC2b fibres in myosteatotic muscles that accompanied with fibre atrophy (*Figures* *6* and *S8*). Previously, we demonstrated that RORα increased the expression of mitochondrial antioxidant enzymes such as superoxide dismutase 2 (SOD2) and Gpx1,^26^ and RORα controlled mitochondrial dynamics by inducing mitochondrial fission proteins, such as Bnip3 and phospho‐Drp1 in the liver.^16^ These observations suggest that RORα may function in restoring mitochondrial phenotypes in MyHC2b fibres. Together, our results support versatile roles of RORα in programming distinct types of fibres by controlling mitochondrial phenotypes, which leads to maintenance of muscle homeostasis.

Skeletal muscle fibre type can have a profound impact on muscle diseases, including muscular dystrophies and sarcopenia.^S1^ In the current study, we observed a significant increase in the number of oxidative type 2a fibres and lipid accumulation in the red region of mouse GA muscles after HFD feeding, similar to observations by others in mice^5^ (*Figure* *1* *B*,*D*). An increase in the cross‐sectional area of oxidative fibres and an accumulation of lipids in oxidative fibres was observed in the vastus lateralis muscle of elderly persons.^S2^ ^,^ ^S3^ These results indicate that oxidative fibre type‐specific distributions of lipids may result in malfunction of skeletal muscle in myosteatosis. In contrast, sarcopenia, a condition with diminished skeletal muscle mass and strength, results from selective atrophy of type 2b fibres.^S4^ Sarcopenia is also considered as a negative prognostic factor in patients with NAFLD.^S5^ ^,^ ^S6^ Interestingly, the level of RORα expression was higher in type 2b than in type 2a muscle fibres in normal‐diet‐fed mice (*Figure* *S9*). The expression of RORα in type 2b muscle fibres was slightly reduced in myosteatotic muscle but was restored when the symptoms of myosteatosis were improved (*Figures* *1* *C* and *6* *A*). These results suggest a role for RORα in type 2b muscle fibres, which may also contribute to protection against or improvement of sarcopenia. Identification of fibre‐specific roles for RORα in different muscle fibre types will assist our understanding of the pathophysiology of muscle diseases, including myosteatosis and sarcopenia. However, a limitation of this study could be a lack of relevance to human muscle diseases, due to a limited number of specimens and technical difficulties in analysing human samples with heterogeneous fibre distributions. Thus, an increased understanding of the fibre specificity of human myosteatosis through future studies could contribute to more effective therapeutic strategies for muscle disease.

Muscle fat is found under two forms: IMCLs and intermuscular adipose tissue (IMAT). The IMCL specifically refers to TGs accumulated within muscle fibres, whereas the IMAT denotes genuine adipose tissue in the intermuscular space along with muscle bundles.^S7^ Elevated levels of both IMCL and IMAT have been shown to augment the susceptibility to metabolic disorders such as obesity and ageing.^S7^ ^,^ ^S8^ Further, the level of accumulated IMCL was closely correlated with insulin resistance in type 2 diabetes patients.^S9^ Because RORα suppresses the accumulation of IMCL as shown in this study, we speculate that RORα restores insulin sensitivity in experimental NAFLD animals as well as patients with diabetes. Indeed, Chai et al. reported that administration of an RORα agonist improved insulin resistance in HFD‐fed mice model.^S10^ Meanwhile, the accumulation of IMAT was not seen in the GA muscles of HFD‐fed mouse model in this study. Because RORα has been shown to inhibit adipocyte differentiation in mouse 3T3‐L1 fibroblasts, it will be interesting to examine whether it affects differentiation and/or accumulation of IMAT.^S11^ ^,^ ^S12^

Thus far, the majority of research conducted in the field of muscle‐related diseases has focused on augmenting muscle mass, particularly for managing sarcopenia. Therapeutic interventions, such as testosterone and selective androgen receptor modulators, are common therapeutic treatments for patients with sarcopenia.^37^ Recently, efforts to enhance muscle function have focused on improving muscle quality, rather than muscle mass, which is determined by various factors including muscle fibre composition, intramuscular fat, muscle aerobic capacity and muscle fibrosis.^38^, ^39^ In the present study, administration of the RORα activator, JC1‐40, reduced intramuscular lipids and restored mitochondrial function in GA muscle in a myosteatosis mouse model, which indicates that RORα activators have potential as therapeutic agents for ameliorating myosteatosis. Furthermore, JC1‐40 administration significantly reduced lipid accumulation and inflammation in the liver, leading to improvement of NAFLD in a mouse model of HFD‐induced NAFLD.^18^, ^25^, ^26^ Together, these observations in the liver and those in muscle obtained in the current study suggest that RORα activators have the potential to act as therapeutic agents for ameliorating NAFLD‐associated myosteatosis.

## Conflict of interest statement

The authors declare that they have no conflicts of interest.

## Supporting information


**Table S1.** Antibodies used in the present investigation.
**Table S2.** Oligonucleotide sequences used in the present investigation.
**Figure S1.** Liver histology visualized by H&E and Oil Red O staining and ALT levels of NAFLD‐associated myosteatosis model.
**Figure S2.** Lipid accumulation in MyHC2a fibres of soleus tissues of HFD‐fed mice.
**Figure S3.** The mRNA expression of Myh7 and Myh2 in ROR*α*‐overexpressed C2C12 cells with palmitic acids treatment.
**Figure S4.** The fluorescence of LipidTOX in ROR*α* ligands‐treated or ROR*α*‐overexpressed C2C12 cells with palmitic acids treatment.
**Figure S5.** Muscular ROR*α* enhances the oxidative function of mitochondria.
**Figure S6.** The mRNA expression of PGC‐1*α* in ROR*α*‐overexpressed or JC1‐40‐treated C2C12 cells.
**Figure S7.** ROR*α* overexpression enhances the expression of TFAM and COX4 and SDH activity in red region of GA of mice with myosteatosis.
**Figure S8.** ROR*α* overexpression alleviates fatty atrophy and recovered the expression of MyHC2b.
**Figure S9.** The expression level of ROR*α* in white region and red region of GA tissues.

## References

[1] Li C‐w , Yu K , Shyh‐Chang N , Jiang Z , Liu T , Ma S , et al. Pathogenesis of sarcopenia and the relationship with fat mass: descriptive review. J Cachexia Sarcopenia Muscle 2022;13:781–794. 35106971 10.1002/jcsm.12901 PMC8977978

[2] Baskin Kedryn K , Winders Benjamin R , Olson EN . Muscle as a “mediator” of systemic metabolism. Cell Metab 2015;21:237–248. 25651178 10.1016/j.cmet.2014.12.021 PMC4398026

[3] Schiaffino S , Reggiani C . Fiber types in mammalian skeletal muscles. Physiol Rev 2011;91:1447–1531. 22013216 10.1152/physrev.00031.2010

[4] He J , Watkins S , Kelley DE . Skeletal muscle lipid content and oxidative enzyme activity in relation to muscle fiber type in type 2 diabetes and obesity. Diabetes 2001;50:817–823. 11289047 10.2337/diabetes.50.4.817

[5] Mastrocola R , Collino M , Nigro D , Chiazza F , D'Antona G , Aragno M , et al. Accumulation of advanced glycation end‐products and activation of the SCAP/SREBP lipogenetic pathway occur in diet‐induced obese mouse skeletal muscle. PLoS ONE 2015;10:e0119587. 25750996 10.1371/journal.pone.0119587 PMC4353621

[6] Lazarus JV , Mark HE , Anstee QM , Arab JP , Batterham RL , Castera L , et al. Advancing the global public health agenda for NAFLD: a consensus statement. Nat Rev Gastroenterol Hepatol 2022;19:60–78. 34707258 10.1038/s41575-021-00523-4

[7] Hsieh YC , Joo SK , Koo BK , Lin HC , Lee DH , Chang MS , et al. Myosteatosis, but not sarcopenia, predisposes NAFLD subjects to early steatohepatitis and fibrosis progression. Clin Gastroenterol Hepatol 2023;21:388–397. 35101634 10.1016/j.cgh.2022.01.020

[8] Nachit M , Kwanten WJ , Thissen JP , Op De Beeck B , Van Gaal L , Vonghia L , et al. Muscle fat content is strongly associated with NASH: a longitudinal study in patients with morbid obesity. J Hepatol 2021;75:292–301. 33865909 10.1016/j.jhep.2021.02.037

[9] Nachit M , De Rudder M , Thissen JP , Schakman O , Bouzin C , Horsmans Y , et al. Myosteatosis rather than sarcopenia associates with non‐alcoholic steatohepatitis in non‐alcoholic fatty liver disease preclinical models. J Cachexia Sarcopenia Muscle 2021;12:144–158. 33244884 10.1002/jcsm.12646 PMC7890270

[10] Lipina C , Hundal HS . Lipid modulation of skeletal muscle mass and function. J Cachexia Sarcopenia Muscle 2017;8:190–201. 27897400 10.1002/jcsm.12144 PMC5377414

[11] Kim J‐Y , Hickner RC , Cortright RL , Dohm GL , Houmard JA . Lipid oxidation is reduced in obese human skeletal muscle. Am J Physiol Endocrinol Metab 2000;279:E1039–E1044. 11052958 10.1152/ajpendo.2000.279.5.E1039

[12] Dominy JE , Puigserver P . Mitochondrial biogenesis through activation of nuclear signaling proteins. Cold Spring Harb Perspect Biol 2013;5:a015008. 23818499 10.1101/cshperspect.a015008 PMC3685894

[13] Gumucio JP , Qasawa AH , Ferrara PJ , Malik AN , Funai K , McDonagh B , et al. Reduced mitochondrial lipid oxidation leads to fat accumulation in myosteatosis. FASEB J 2019;33:7863–7881. 30939247 10.1096/fj.201802457RR PMC6593892

[14] Roden M . Muscle triglycerides and mitochondrial function: possible mechanisms for the development of type 2 diabetes. Int J Obes (Lond) 2005;29:S111–S115. 16385762 10.1038/sj.ijo.0803102

[15] Cook DN , Kang HS , Jetten AM . Retinoic acid‐related orphan receptors (RORs): regulatory functions in immunity, development, circadian rhythm, and metabolism. Nucl Receptor Res 2015;2:101185. 26878025 10.11131/2015/101185 PMC4750502

[16] Kim H‐J , Han Y‐H , Na H , Kim J‐Y , Kim T , Kim H‐J , et al. Liver‐specific deletion of RORα aggravates diet‐induced nonalcoholic steatohepatitis by inducing mitochondrial dysfunction. Sci Rep 2017;7:16041. 29167529 10.1038/s41598-017-16077-y PMC5700103

[17] Kim H‐J , Han Y‐H , Kim J‐Y , Lee M‐O . RORα enhances lysosomal acidification and autophagic flux in the hepatocytes. Hepatol Commun 2021;5:2121–2138. 34558854 10.1002/hep4.1785 PMC8631090

[18] Han Y‐H , Kim H‐J , Na H , Nam M‐W , Kim J‐Y , Kim J‐S , et al. RORα induces KLF4‐mediated M2 polarization in the liver macrophages that protect against nonalcoholic steatohepatitis. Cell Rep 2017;20:124–135. 28683306 10.1016/j.celrep.2017.06.017

[19] Kim K , Boo K , Yu YS , Oh SK , Kim H , Jeon Y , et al. RORα controls hepatic lipid homeostasis via negative regulation of PPARγ transcriptional network. Nat Commun 2017;8:162. 28757615 10.1038/s41467-017-00215-1 PMC5534431

[20] Jarvis CI , Staels B , Brugg B , Lemaigre‐Dubreuil Y , Tedgui A , Mariani J . Age‐related phenotypes in the staggerer mouse expand the RORα nuclear receptor's role beyond the cerebellum. Mol Cell Endocrinol 2002;186:1–5. 11850116 10.1016/s0303-7207(01)00668-2

[21] Lau P , Bailey P , Dowhan DH , Muscat GEO . Exogenous expression of a dominant negative RORα1 vector in muscle cells impairs differentiation: RORα1 directly interacts with p300 and MyoD. Nucleic Acids Res 1999;27:411–420. 9862959 10.1093/nar/27.2.411 PMC148194

[22] Lau P , Nixon SJ , Parton RG , Muscat GEO . RORα regulates the expression of genes involved in lipid homeostasis in skeletal muscle cells: caveolin‐3 and CPT‐1 are direct targets of ROR. J Biol Chem 2004;279:36828–36840. 15199055 10.1074/jbc.M404927200

[23] Nohara K , Mallampalli V , Nemkov T , Wirianto M , Yang J , Ye Y , et al. Nobiletin fortifies mitochondrial respiration in skeletal muscle to promote healthy aging against metabolic challenge. Nat Commun 2019;10:3923. 31462679 10.1038/s41467-019-11926-y PMC6713763

[24] Hirai T , Nomura K , Ikai R , Nakashima K‐i , Inoue M . Baicalein stimulates fibroblast growth factor 21 expression by up‐regulating retinoic acid receptor‐related orphan receptor α in C2C12 myotubes. Biomed Pharmacother 2019;109:503–510. 30399586 10.1016/j.biopha.2018.10.154

[25] Kim EJ , Yoon YS , Hong S , Son HY , Na TY , Lee MH , et al. Retinoic acid receptor‐related orphan receptor α‐induced activation of adenosine monophosphate‐activated protein kinase results in attenuation of hepatic steatosis. Hepatology 2012;55:1379–1388. 22183856 10.1002/hep.25529

[26] Han YH , Kim HJ , Kim EJ , Kim KS , Hong S , Park HG , et al. RORα decreases oxidative stress through the induction of SOD2 and GPx1 expression and thereby protects against nonalcoholic steatohepatitis in mice. Antioxid Redox Signal 2014;21:2083–2094. 24597775 10.1089/ars.2013.5655 PMC4215383

[27] Rabey KN , Satkunam L , Webber CA , Hocking JC . Isolated fatty infiltration of the gastrocnemius medial head, a cadaveric case study. Hum Pathol: Case Rep 2021;23:200480.

[28] Stark DA , Coffey NJ , Pancoast HR , Arnold LL , Walker JP , Vallée J , et al. Ephrin‐A3 promotes and maintains slow muscle fiber identity during postnatal development and reinnervation. J Cell Biol 2015;211:1077–1091. 26644518 10.1083/jcb.201502036 PMC4674275

[29] Dimauro I , Antonioni A , Mercatelli N , Grazioli E , Fantini C , Barone R , et al. The early response of αB‐crystallin to a single bout of aerobic exercise in mouse skeletal muscles depends upon fiber oxidative features. Redox Biol 2019;24:101183. 30974319 10.1016/j.redox.2019.101183 PMC6454247

[30] Rantanen A , Jansson M , Oldfors A , Larsson N‐G . Downregulation of Tfam and mtDNA copy number during mammalian spermatogenesis. Mamm Genome 2001;12:787–792. 11668394 10.1007/s00335-001-2052-8

[31] Slavin MB , Memme JM , Oliveira AN , Moradi N , Hood DA . Regulatory networks coordinating mitochondrial quality control in skeletal muscle. Am J Physiol Cell Physiol 2022;322:C913–C926. 35353634 10.1152/ajpcell.00065.2022

[32] Theilen NT , Kunkel GH , Tyagi SC . The role of exercise and TFAM in preventing skeletal muscle atrophy. J Cell Physiol 2017;232:2348–2358. 27966783 10.1002/jcp.25737 PMC5444986

[33] Saleem A , Hood DA . Acute exercise induces tumour suppressor protein p53 translocation to the mitochondria and promotes a p53–Tfam–mitochondrial DNA complex in skeletal muscle. J Physiol 2013;591:3625–3636. 23690562 10.1113/jphysiol.2013.252791 PMC3731618

[34] Kim E‐J , Choi Y‐K , Han Y‐H , Kim H‐J , Lee I‐K , Lee M‐O . RORα suppresses proliferation of vascular smooth muscle cells through activation of AMP‐activated protein kinase. Int J Cardiol 2014;175:515–521. 25017905 10.1016/j.ijcard.2014.06.043

[35] Picard M , Hepple RT , Burelle Y . Mitochondrial functional specialization in glycolytic and oxidative muscle fibers: tailoring the organelle for optimal function. Am J Physiol Cell Physiol 2011;302:C629–C641. 22031602 10.1152/ajpcell.00368.2011

[36] Mishra P , Varuzhanyan G , Pham Anh H , Chan DC . Mitochondrial dynamics is a distinguishing feature of skeletal muscle fiber types and regulates organellar compartmentalization. Cell Metab 2015;22:1033–1044. 26603188 10.1016/j.cmet.2015.09.027 PMC4670593

[37] Dennison EM , Sayer AA , Cooper C . Epidemiology of sarcopenia and insight into possible therapeutic targets. Nat Rev Rheumatol 2017;13:340–347. 28469267 10.1038/nrrheum.2017.60 PMC5444517

[38] McGregor RA , Cameron‐Smith D , Poppitt SD . It is not just muscle mass: a review of muscle quality, composition and metabolism during ageing as determinants of muscle function and mobility in later life. Longev Healthspan 2014;3:9. 25520782 10.1186/2046-2395-3-9 PMC4268803

[39] Selvais CM , Davis‐López de Carrizosa MA , Nachit M , Versele R , Dubuisson N , Noel L , et al. AdipoRon enhances healthspan in middle‐aged obese mice: striking alleviation of myosteatosis and muscle degenerative markers. J Cachexia Sarcopenia Muscle 2023;14:464–478. 36513619 10.1002/jcsm.13148 PMC9891981

