# Supplementary material for: RORα–GABP–TFAM axis alleviates myosteatosis with fatty atrophy through reinforcement of mitochondrial capacity
Source: J Cachexia Sarcopenia Muscle. 2024 Jan 25;15(2):615–30. doi: 10.1002/jcsm.13432 (PMC10995264; doi:10.1002/jcsm.13432)
Supplement: Supplementary file 1 — Table S1. Antibodies used in the present investigation. Table S2. Oligonucleotide sequences used in the present investigation. Figure S1. Liver histology visualized by H&E and Oil Red O staining and ALT levels of NAFLD‐associated myosteatosis model. Figure S2. Lipid accumulation in MyHC2a fibres of soleus tissues of HFD‐fed mice. Figure S3. The mRNA expression of Myh7 and Myh2 in RORα‐overexpressed C2C12 cells with palmitic acids treatment. Figure S4. The fluorescence of LipidTOX in RORα ligands‐treated or RORα‐overexpressed C2C12 cells with palmitic acids treatment. Figure S5. Muscular RORα enhances the oxidative function of mitochondria. Figure S6. The mRNA expression of PGC‐1α in RORα‐overexpressed or JC1‐40‐treated C2C12 cells. Figure S7. RORα overexpression enhances the expression of TFAM and COX4 and SDH activity in red region of GA of mice with myosteatosis. Figure S8. RORα overexpression alleviates fatty atrophy and recovered the expression of MyHC2b. Figure S9. The expression level of RORα in white region and red region of GA tissues. [file JCSM-15-615-s001.pdf]

Supporting Information

RORα-GABP-TFAM axis alleviates myosteatosis with fatty atrophy through reinforcement of mitochondrial capacity

Hyeon-Ji Kim[1,2], Sang-Heon Lee[1], Cheolhee Jeong[1], Yong-Hyun Han[3], and Mi-Ock Lee[1,2,4]

[1]College of Pharmacy, Seoul National University, Seoul, Korea; [2]Research Institute of Pharmaceutical Sciences, Seoul, Korea; [3]College of Pharmacy, Kangwon National University, Chuncheon, South Korea; [4]Bio-MAX Institute, Seoul National University, Seoul, Korea

Address correspondence to: Mi-Ock Lee Ph. D., College of Pharmacy, Seoul National University, 1 Gwanak-ro, Gwanak-gu, Seoul 08826, Korea. E-mail: molee@snu.ac.kr

Supplementary Methods

Assessment of oxygen consumption rate

To assess oxygen consumption rate (OCR), C2C12 cells were plated at 2.5×10^4 cells/well in DMEM containing 10% FBS. After 24 h of seeding, Ad-GFP or Ad-GFP-RORα particles (multiplicity of infection = 100) were added into the culture media. At 24 h after virus infection, media were exchanged to differentiation medium and cells were further incubated for 48 h. OCR was analyzed using the Seahorse XF24 Extracellular Flux Analyzer (Seahorse Bioscience, North Billerica, MA, USA). To determine basal and maximal respiration, 2 μM oligomycin, 5 μM carbonyl cyanide-4-(trifluoromethoxy)phenylhydrazone (FCCP), an inducer of maximal respiration, and 1 μM antimycin A/rotenone were added to the media. The basal OCR was calculated as the OCR baseline levels before FCCP injection minus the average of three OCR levels after antimycin A/rotenone injection (non-mitochondrial respiration). Maximal OCR was produced by subtracting non-mitochondrial respiration from the OCR levels after FCCP injection.

Supplementary Table 1. Antibodies used in the present investigation.

Purpose	Protein	Primary Ab	Titer	Secondary Ab	Titer
IHC	MyHC2a	DSHB (SC-71)	1:100	Polink-2 Plus HRP Mouse DAB Detection Kit (ORIGENE, D37-18)	1:100
	COX4	Santa Cruz Biotechnology (sc-517553)	1:100	Polink-2 Plus HRP Mouse DAB Detection Kit (ORIGENE, D37-18)	1:100
	ADFP/PLIN2	Invitrogen (PA1-16972)	1:100	Polink-2 Plus HRP Rabbit DAB Detection Kit (ORIGENE, D39-6)	1:100
	RORα	Abcam (ab256799)	1:100	Polink-2 Plus HRP Rabbit DAB Detection Kit (ORIGENE, D39-6)	1:100
IF	MyHC2a	DSHB (SC-71)	1:100	Goat anti-mouse IgG, Alexa Fluor 405 (Invitrogen, A-31553) Donkey anti-mouse IgG, Alexa Fluor 488 (Invitrogen, A-21202)	1:100
	MyHC2b	DSHB (BF-F3)	1:100	Goat anti-mouse IgM, Alexa Fluor 555 (Invitrogen, A-21426)	1:100
	MyHC1	DSHB (BA-F8)	1:100	Donkey anti-mouse IgG, Alexa Fluor 488 (Invitrogen, A-21202)	1:100
	SDHA	Santa Cruz Biotechnology (sc-390381)	1:100	Donkey anti-mouse IgG, Alexa Fluor 488 (Invitrogen, A-21202)	1:100
	TFAM	Santa Cruz Biotechnology (sc-28200)	1:100	Donkey anti-rabbit IgG, Alexa Fluor 555 (Invitrogen, A-31572)	1:100
	COX4	Santa Cruz Biotechnology (sc-58348)	1:100	Donkey anti-mouse IgG, Alexa Fluor 488 (Invitrogen, A-21202)	1:100
	RORα	Abcam (ab256799)	1:100	Chicken anti-rabbit IgG, Alexa Fluor 488 (Invitrogen, A-21441)	1:100
WB	OXPHOS	Invitrogen (458099)	1:5000	Goat anti-mouse IgG HRP (Invitrogen, G-21040)	1:5000
	TFAM	Santa Cruz Biotechnology (sc-28200)	1:5000	Goat anti-rabbit IgG HRP (Invitrogen, G-21234)	1:5000
	NRF1	Santa Cruz Biotechnology (sc-33771)	1:10000	Goat anti-rabbit IgG HRP (Invitrogen, G-21234)	1:10000
	GABPα	Santa Cruz Biotechnology (sc-28312)	1:2000	Goat-anti-mouse IgG HRP (Invitrogen, G-21040)	1:2000
	RORα	Abcam (ab256799)	1:2000	Goat anti-rabbit IgG HRP (Invitrogen, G-21234)	1:2000
	β-actin	Santa Cruz Biotechnology (sc-47778)	1:5000	Goat anti-mouse IgG HRP (Invitrogen, G-21040)	1:5000
	α-tubulin	Millipore (05-829)	1:10000	Goat anti-mouse IgG HRP (Invitrogen, G-21040)	1:10000
	Hsp60	Abcam (ab45134)	1:10000	Goat anti-rabbit IgG HRP (Invitrogen, G-21234)	1:10000
ChIP	GABPα	Santa Cruz Biotechnology (sc-28312)			
	histone 3 (acetyl K27)	Abcam (ab4729)			
	RORα	Abcam (ab256799)			

Supplementary Table 2. Oligonucleotide sequences used in the present investigation.

Purpose	Gene (mouse)	Accession number	Nucleotide sequence	
qRT-PCR	Myh7	NM_080728.3	Sense	5'-CCAAGGGCCTGAATGAGGAG-3'
		NM_001361607.1	Antisense	5'-GCAAAGGCTCCAGGTCTGAG-3'
	Myh2	NM_001039545.2	Sense	5'-CCAAGAAAGGTGCCAAGAAG-3'
			Antisense	5'-CGGGAGTCTTGGTTTCATTG-3'
	Myh4	NM_010855.3	Sense	5'-CCGAGCAAGAGCTACTGGA-3'
			Antisense	5'-TGTTGATGAGGCTGGTGTTC-3'
	RORα	NM_013646.2	Sense	5'-TTTCAGGAGAAGTCAGCAGAG-3'
		NM_001289916.1	Antisense	5'-TCTGCTGGTCCGATCAATCAA-3'
	18s rRNA	NR_003278.3	Sense	5'-GTAACCCGTTGAACCCCATT-3'
			Antisense	5'-CCATCCAATCGGTAGTAGCG-3'
ChIP	NRF2 binding sequence in TFAM promoter		Sense	5'-GCACAGCACCTCCTGTCCTC-3'
			Antisense	5'-GTCCGGTTCGTCTCACGCAA-3
	Putative RORE in NRF2α promoter		Sense	5'-AAACAACGTCAAGGCCAAG-3'
			Antisense	5'-TTCAAGGGTCTGACGAGGAG-3
Knockdown	si-GL3		Sense	5'-CUUACGCUGAGUACUUCGA-3'
			Antisense	5'-UCGAAGUACUCAGCGUAAG-3
	si-RORα		Sense	5'-GCAGAGAGACAGCUUGUACGC-3'
			Antisense	5'-GCGUACAAGCUGUCUCUCUGC-3

Supplementary Figures

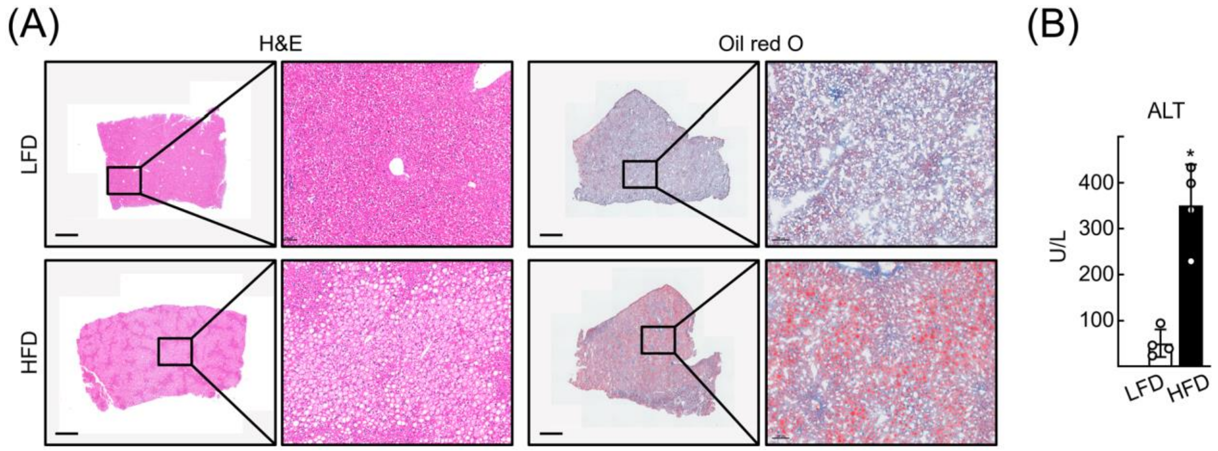

Supplementary Figure 1. Liver histology visualized by H&E and Oil Red O staining and ALT levels of NAFLD-associated myosteatosis model.
Seven week-old mice were fed with either low-fat diet (LFD) or high-fat diet (HFD) for 20 weeks. (A) H&E and Oil red O staining of liver sections. Scale bar: 1 mm. (B) Serum alanine aminotransferase (ALT) activities were measured by standard clinical chemistry assays at the end of experiments. $^{*}P < 0.05$ *vs* LFD-fed mice (n=4).

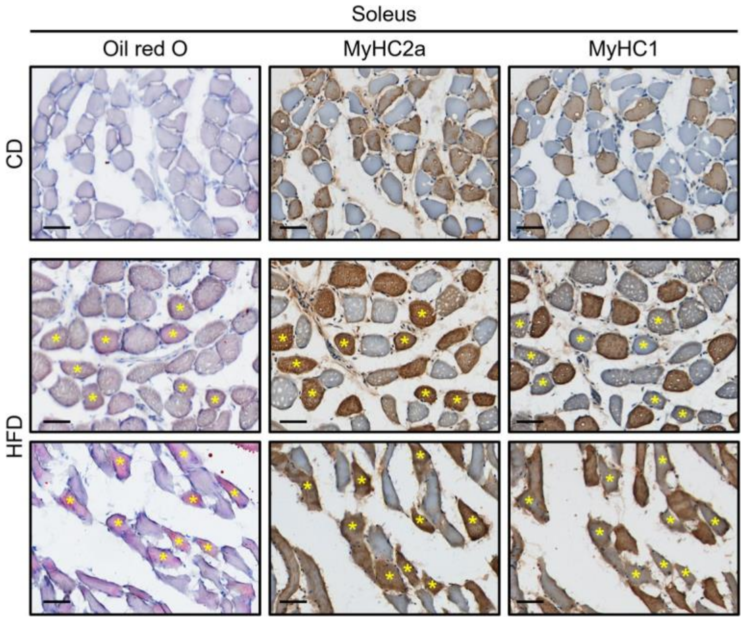

Supplementary Figure 2. Lipid accumulation in MyHC2a fibers of soleus tissues of HFD-fed mice.
Nine week-old mice were fed with either chow diet (CD) or high-fat diet (HFD) for 16 weeks. Oil red O staining and immunohistochemical staining for MyHC2a and MyHC1 of soleus tissues sections. Representative images are shown. Yellow stars indicate oxidative MyHC2a fibers with intensive lipid infiltration. Scale bar: 50 μm.

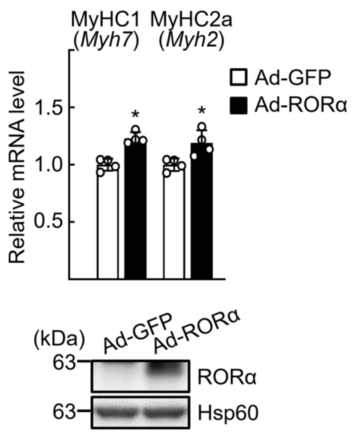

Supplementary Figure 3. The mRNA expression of Myh7 and Myh2 in RORα-overexpressed C2C12 cells with palmitic acids treatment.

Ad-GFP or Ad-RORα-transduced C2C12 cells were incubated in differentiation medium containing 0.1 mM palmitic acid conjugated with bovine serum albumin for 7 days. Total RNA was isolated and mRNA levels of the indicated genes were measured by qRT-PCR. Expression levels of RORα in adenovirus-transduced C2C12 cells were analyzed by western blotting. $^{*}P < 0.05$ *vs* Ad-GFP-infused C2C12 cells (n=4).

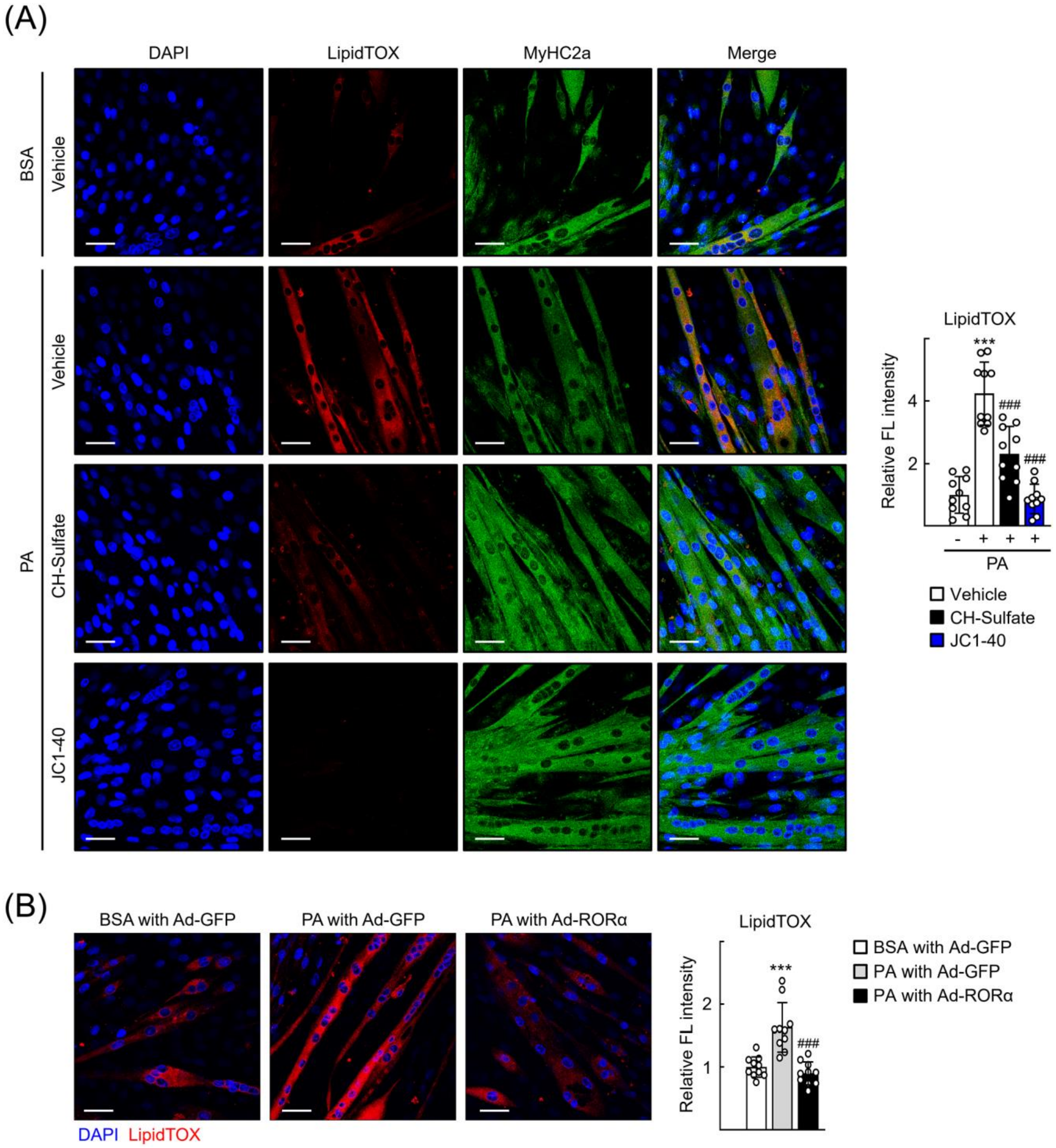

Supplementary Figure 4. The fluorescence of LipidTOX in RORα ligands-treated or RORα-overexpressed C2C12 cells with palmitic acids treatment.

(A) C2C12 cells were incubated in differentiation medium with 0.1 mM palmitic acid (PA) conjugated with bovine serum albumin (BSA), and treated with cholesterol sulfate (CH-Sulfate) or JC1-40. After 7 days, immunostaining was performed for staining of MyHC2a (green). Myotubes were stained with LipidTOX Red for visualization of neutral lipids, and examined by confocal microscopy. Representative images are shown. Data were obtained from three independent experiments and fluorescence intensity was quantified in ten images of each group by using ImageJ. Scale bar: 25 μm. $^{***}P < 0.001$ *vs* Vehicle with BSA and $^{\#\#\#}P < 0.001$ *vs* Vehicle with PA.

(B) Ad-GFP or Ad-RORα-transduced C2C12 cells were incubated in differentiation medium containing 0.1 mM palmitic acid (PA) conjugated with bovine serum albumin (BSA) for 7 days. Myotubes were stained with LipidTOX Red for visualization of neutral lipids, and examined by confocal microscopy. Representative images are shown. Data were obtained from three independent experiments and fluorescence intensity was quantified in ten images of each group by using ImageJ. Scale bar: 25 μm. $^{***}P < 0.001$ *vs* Ad-GFP with BSA and $^{\#\#\#}P < 0.001$ *vs* Ad-GFP with PA.

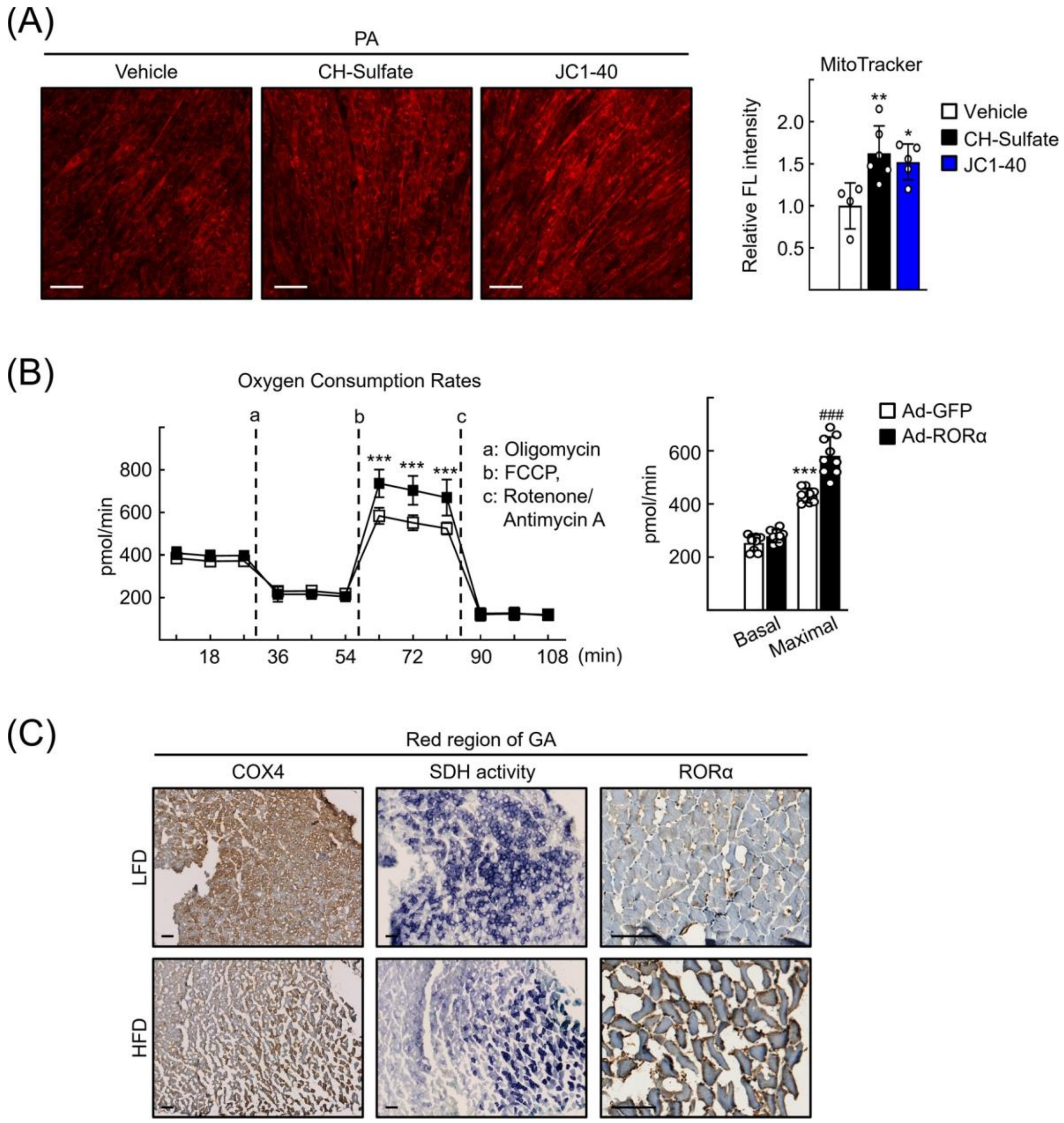

Supplementary Figure 5. Muscular RORα enhances the oxidative function of mitochondria.

(A) C2C12 cells were grown in medium containing 0.1 mM palmitic acid (PA) conjugated with bovine serum albumin and cholesterol sulfate (CH-Sulfate) or JC1-40. After 48 h, cells were stained with MitoTracker Red CMXRos and subjected to confocal microscopy. Data were obtained from three independent experiments and fluorescence intensity was quantified in at least four images of each group using ImageJ. Scale bar: 50 μm. $^{*}P < 0.05$ and $^{**}P < 0.01$ *vs* Vehicle.

(B) The basal oxygen consumption rate (OCR) and uncoupled respiration of Ad-GFP and Ad-RORα-transduced C2C12 cells in media containing 4,500 mg/L of glucose. a, b, and c refer to the time course of adding oligomycin which is a ATPase inhibitor, FCCP, a inducer of uncoupled respiration, and antimycin A/rotenone, respectively. The basal OCR and maximal respiration were calculated based on data in upper panel. The basal OCR was calculated by the OCR baseline levels before FCCP injection minus the average of three OCR levels after antimycin A/rotenone injection (non-mitochondrial respiration). Maximal OCR was produced by subtracting non-mitochondrial respiration from the OCR levels after FCCP injection. $^{***}P < 0.001$ *vs* basal OCR of Ad-GFP-transduced C2C12 cells and $^{\#\#\#}P < 0.001$ *vs* maximal OCR of Ad-GFP-transduced C2C12 cells (n=9).

(C) Expression of COX4 and RORα in the red region of gastrocnemius (GA) sections of the high-fat diet (HFD)-fed mice was visualized by immunohistochemistry. Succinate dehydrogenase (SDH) staining was performed with the GA sections of either low-fat diet (LFD) or HFD-fed mice. Representative images are shown. Scale bar: 100 μm.

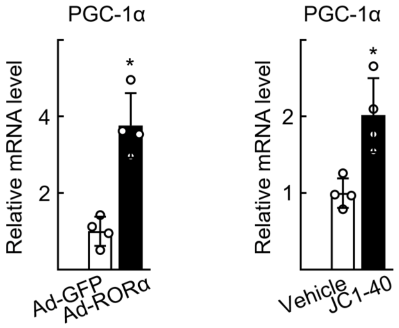

Supplementary Figure 6. The mRNA expression of PGC-1α in RORα-overexpressed or JC1-40-treated C2C12 cells.
Adenovirus-transduced or JC1-40-treated C2C12 cells were incubated in differentiation medium for 48 h. Total RNA was isolated and mRNA level of PGC-1α was measured by qRT-PCR. $^{*}P < 0.05$ *vs* Ad-GFP-transduced or Vehicle-treated C2C12 cells (n=4).

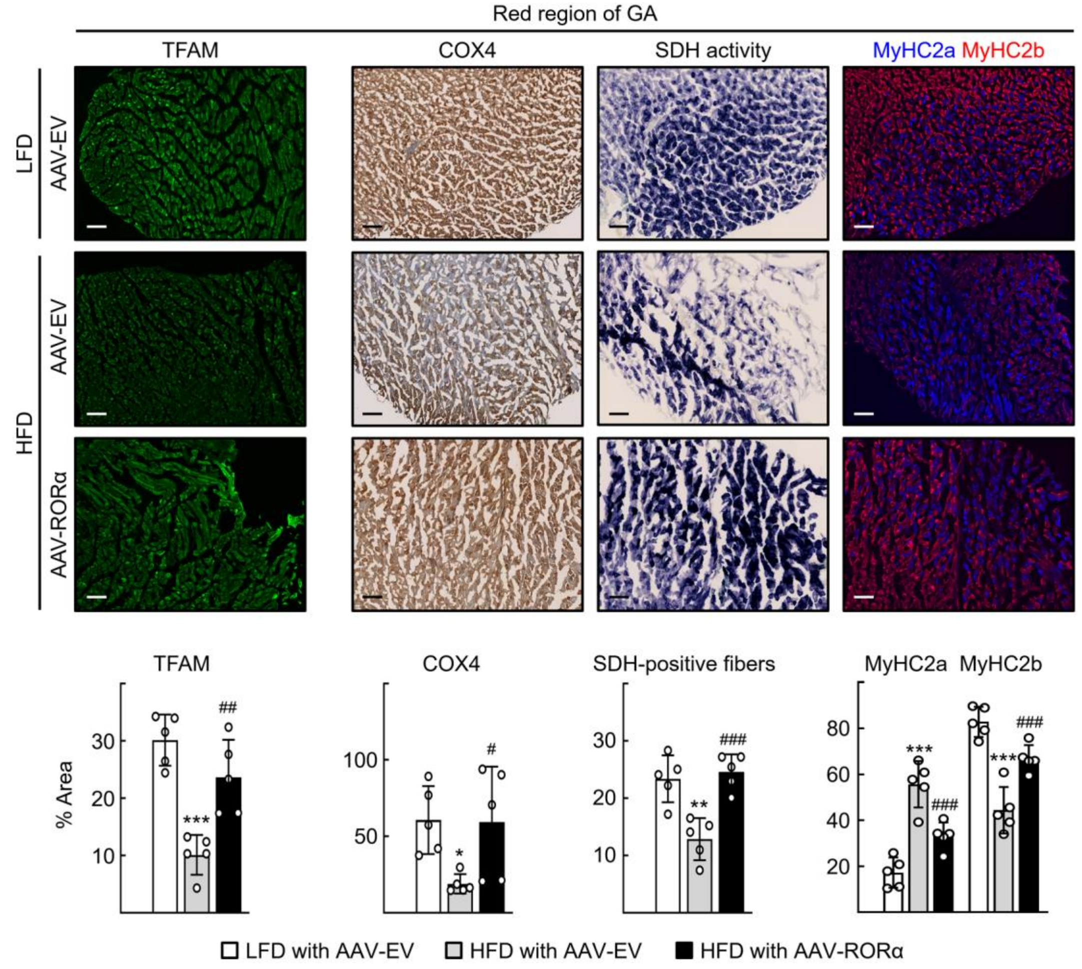

Supplementary Figure 7. RORα overexpression enhances the expression of TFAM and COX4 and SDH activity in red region of GA of mice with myosteatosis.

Mouse gastrocnemius (GA) tissues were subjected to immunostaining for TFAM (green). Expression of COX4 in the red region of GA sections of the HFD-fed and AAV-RORα-treated mouse model was visualized by immunohistochemistry. Succinate dehydrogenase (SDH) staining was performed with the GA sections of the HFD-fed and AAV-RORα-treated mouse model. The red region of GA tissue sections were subjected to immunostaining for MyHC2a (blue) and MyHC2b (red). Tissues were examined by using an automated multimodal tissue analysis system and representative images of are shown. Quantification of TFAM, COX4, SDH-positive fibers, MyHC2a (blue), and MyHC2b (red) was performed by using ImageJ and the occupancy of TFAM, COX4, and SDH-positive fibers in the total GA muscles and the occupancy of MyHC2a and MyHC2b in the red region of GA were expressed as a percentage. Scale bar: 200 μm. $^{*}P < 0.05$, $^{**}P < 0.01$, and $^{***}P < 0.001$ *vs* LFD-fed and AAV-EV-treated mouse (n=5). $^{\#}P < 0.05$, $^{\#\#}P < 0.01$, and $^{\#\#\#}P < 0.001$ *vs* HFD-fed and AAV-EV-treated mouse (n=5).

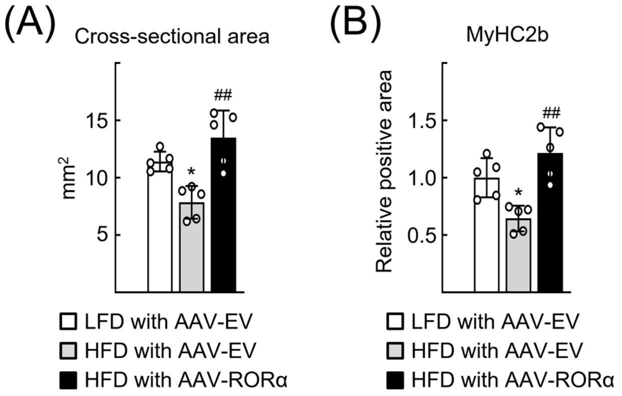

Supplementary Figure 8. RORα overexpression alleviates fatty atrophy and recovered the expression of MyHC2b.

(A) Quantification of cross-sectional area of gastrocnemius was performed by using ImageJ. $^{*}P < 0.05$ *vs* LFD-fed and AAV-EV-treated mouse (n=5). $^{\#\#}P < 0.01$ *vs* HFD-fed and AAV-EV-treated mouse (n=5).

(B) Quantification of MyHC2b fibers was performed by using ImageJ. $^{*}P < 0.05$ *vs* LFD-fed and AAV-EV-treated mouse (n=5). $^{\#\#}P < 0.01$ *vs* HFD-fed and AAV-EV-treated mouse (n=5). (Corresponding to Figure 6A)

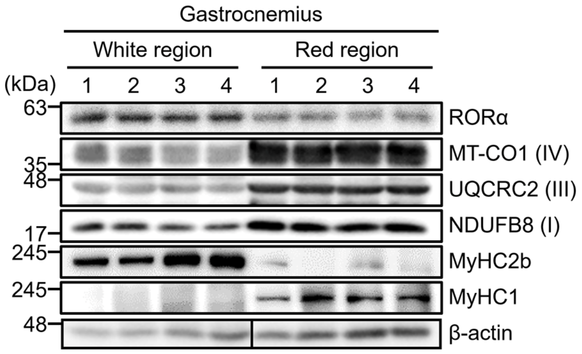

Supplementary Figure 9. The expression level of RORα in white region and red region of GA tissues.
Expression levels of RORα, MyHC2b, MyHC1, and mitochondrial protein in the white region and red region of gastrocnemius tissues of normal-diet-fed mice were analyzed by western blotting. The lane of β-actin was run on the same gel but was noncontiguous.

Supplementary References

S1. Talbot J, Maves L. Skeletal muscle fiber type: using insights from muscle developmental biology to dissect targets for susceptibility and resistance to muscle disease. WIREs Developmental Biology. 2016;5:518-34.

S2. Gueugneau M, Coudy-Gandilhon C, Théron L, Meunier B, Barboiron C, Combaret L, et al. Skeletal Muscle Lipid Content and Oxidative Activity in Relation to Muscle Fiber Type in Aging and Metabolic Syndrome. The Journals of Gerontology: Series A. 2015;70:566-76.

S3. St-Jean-Pelletier F, Pion CH, Leduc-Gaudet J-P, Sgarioto N, Zovilé I, Barbat-Artigas S, et al. The impact of ageing, physical activity, and pre-frailty on skeletal muscle phenotype, mitochondrial content, and intramyocellular lipids in men. Journal of Cachexia, Sarcopenia and Muscle. 2017;8:213-28.

S4. Nilwik R, Snijders T, Leenders M, Groen BBL, van Kranenburg J, Verdijk LB, et al. The decline in skeletal muscle mass with aging is mainly attributed to a reduction in type II muscle fiber size. Experimental gerontology. 2013;48:492-8.

S5. Koo BK, Kim D, Joo SK, Kim JH, Chang MS, Kim BG, et al. Sarcopenia is an independent risk factor for non-alcoholic steatohepatitis and significant fibrosis. Journal of Hepatology. 2017;66:123-31.

S6. Seo JY, Cho EJ, Kim MJ, Kwak M-S, Yang JI, Chung SJ, et al. The relationship between metabolic dysfunction-associated fatty liver disease and low muscle mass in an asymptomatic Korean population. Journal of Cachexia, Sarcopenia and Muscle. 2022;13:2953-60.

S7. Goodpaster BH, Bergman BC, Brennan AM, Sparks LM. Intermuscular adipose tissue in metabolic disease. Nat Rev Endocrinol. 2023;19:285-98.

S8. Laurens C, Moro C. Intramyocellular fat storage in metabolic diseases. Horm Mol Biol Clin Investig. 2016;26:43-52.

S9. Anderwald C, Bernroider E, Krš š ák M, Stingl H, Brehm A, Bischof MG, et al. Effects of Insulin Treatment in Type 2 Diabetic Patients on Intracellular Lipid Content in Liver and Skeletal Muscle. Diabetes. 2002;51:3025-32.

S10. Chai C, Cox B, Yaish D, Gross D, Rosenberg N, Amblard F, et al. Agonist of RORA Attenuates Nonalcoholic Fatty Liver Progression in Mice via Up-regulation of MicroRNA 122. Gastroenterology. 2020;159:999-1014.

S11. Duez H, Duhem C, Laitinen S, Patole PS, Abdelkarim M, Bois-Joyeux B, et al. Inhibition of adipocyte differentiation by RORα. FEBS Letters. 2009;583:2031-6.

S12. Ohoka N, Kato S, Takahashi Y, Hayashi H, Sato R. The orphan nuclear receptor RORalpha restrains adipocyte differentiation through a reduction of C/EBPbeta activity and perilipin gene expression. Mol Endocrinol. 2009;23:759-71.
